# Multi-feature fusion for gene prediction and functional peptide identification

**DOI:** 10.3389/fmicb.2026.1736391

**Published:** 2026-02-06

**Authors:** Chenjing Ma, Qianran Wei, Guohua Wang, Yan Miao, Lei Yuan

**Affiliations:** 1 Department of Hepatobiliary Surgery, The Quzhou Affiliated Hospital of Wenzhou Medical University, Quzhou People's Hospital, Quzhou, Zhejiang, China; 2 College of Computer and Control Engineering, Northeast Forestry University, Harbin, Heilongjiang, China; 3 Faculty of Computing, Harbin Institute of Technology, Harbin, Heilongjiang, China

**Keywords:** ACP, AMPs, deep learning, functional peptide prediction, gene prediction

## Abstract

Anticancer peptides (ACPs) have demonstrated potent antitumor activity and low toxicity, offering considerable potential in cancer therapeutics. Meanwhile, antimicrobial peptides (AMPs)serve as key components of the innate immune defense system. Owing to their broad-spectrum antimicrobial activity and low propensity for inducing resistance, AMPs have attracted considerable attention in the fields of infection control and immunotherapy. Accurate identification of ACPs and AMPs is critical for the discovery of novel therapeutic agents. However, wet-lab identification is often time-consuming, costly, and inefficient, falling short of the demands for highthroughput drug screening. Furthermore, existing computational methods exhibit limitations in feature representation and cross-task prediction capability. To address these challenges, a tool for functional peptide prediction is proposed, namely GP2FI, which consists of two sequential stages: a gene prediction model (MHA-preconv) and a functional peptide identification model (FuncPred-CB). MHA-preconv integrates CNNs with Transformer encoder layers to form a two-stage deep architecture, effectively capturing both local sequence patterns and long-range dependencies. Based on the coding regions identified by MHA-preconv, FuncPred-CB incorporates a pre-trained BERT language model to automatically extract contextual semantic features from amino acid sequences. Experimental results on multiple benchmark datasets demonstrate that MHA-preconv and GP2FI consistently outperforms the state-of-the-art methods in terms of accuracy and other performance metrics.The code for the GP2FI can be found at https://github.com/ma999-mxl/maLBX.git.

## 1 Introduction

Functional peptides, particularly anticancer peptides (ACPs) and antimicrobial peptides (AMPs), have emerged as prominent research topics in recent years due to their critical roles in cancer therapy and immune defense. ACPs exhibit remarkable anti-tumor potential by selectively targeting cancer cells through unique membrane-disruptive mechanisms. AMPs, on the other hand, are widely distributed in living organisms and possess broad-spectrum antimicrobial activity with low risk of resistance development. They have been extensively applied in medicine, food safety, and agriculture. However, the experimental identification of such functional peptides is time-consuming and costly, which significantly hinders their large-scale development and practical application.

With the rapid development of artificial intelligence technologies, sequence-based functional peptide prediction has emerged as a feasible and efficient alternative approach. This predictive process generally involves two key stages: first, deep learning methods are employed to accurately identify open reading frames (ORFs) from raw genomic sequences; second, the predicted gene sequences are translated into protein sequences, which are subsequently analyzed by machine learning or deep learning models to identify potential ACPs or AMPs. Therefore, constructing an end-to-end framework that integrates efficient gene prediction and functional peptide identification is of great importance for the discovery of novel functional peptides and the advancement of precision medicine and anti-infective therapeutics.

For gene prediction, a variety of algorithms have been proposed to identify ORFs with protein-coding potential in genomic sequences. These methods can generally be categorized into three main types: statistical learning-based, traditional machine learning-based, and deep learning-based approaches. Early tools such as Prodigal (Hyatt et al., 2010) employed Hidden Markov Models (HMMs) combined with statistical scoring schemes to rank and evaluate ORFs. While these approaches are computationally efficient, they often struggle to capture complex sequence patterns and long-range dependencies (Larsen and Krogh, 2003; Delcher et al., 2007; Kelley et al., 2011). Traditional machine learning-based methods, such as Orphelia (Hoff et al., 2009) and MGC (El Allali and Rose, 2013), integrated neural networks with discriminative classifiers. MetaGUN (Liu et al., 2013), MetaGeneAnnotator (Noguchi et al., 2008), and mRMR-SVM (Al-Ajlan and El Allali, 2018a) utilized Support Vector Machines (SVMs) for gene classification. FragGeneScan (Rho et al., 2010) combined sequencing error models with codon usage preferences, enhancing its robustness on low-quality data. Additionally, an m5C-Seq (Abbas et al., 2024) model—an ensemble-learning approach for predicting RNA 5-methylcytosine modification sites—and an ML (Abbas et al., 2025a) model designed for rare genetic diseases, which leverages machine learning to handle high-dimensional genomic data in a manner that deviates from the traditional single-gene prediction paradigm, have been introduced. Nevertheless, these methods still exhibit limitations in deep feature extraction and modeling of sequence-level dependencies. In response, a growing number of deep learning-based models have been established in recent years. Meta-MFDL (Zhang et al., 2017) employs a multi-layer stacked architecture for feature extraction and classification. CNN-MGP (Al-Ajlan and El Allali, 2018b) constructs a multi-branch CNN ensemble for gene prediction. CNN-RAI (Karagöz and Nalbantoglu, 2021) leverages k-mer features in a CNN framework. Although these models improve prediction accuracy, gene prediction commonly faces challenges such as short read lengths, incomplete sequences, and fragmentation, leading to loss of sequence information. Additionally, limitations in capturing global sequence dependencies further increase the difficulty of accurate gene identification.

For functional peptide identification, research has primarily focused on the independent prediction of two major categories: ACPs and AMPs. For ACP prediction, various models have been developed by transforming peptide sequences into numerical representations and applying classification algorithms. Representative methods include ACP-DRL (Xu et al., 2024), PEPred-Suite (Wei et al., 2019), ACPred-Fuse (Rao et al., 2019), iACP-DRLF (Lv et al., 2021), AntiCP2.0 (Agrawal et al., 2020), ACP-check (Zhu et al., 2022), and ACP-BC (Sun et al., 2023), which utilize dipeptide composition, deep representation learning, Bi-LSTM architectures, or multi-channel data augmentation strategies for modeling. In the field of AMP prediction, the construction of large-scale AMP databases such as CAMP (Waghu et al., 2015), APD3 (Wang, 2004), dbAMP (Jhong et al., 2018), and DRAMP 2.0 (Kang et al., 2019) has provided essential data resources for computational model development. Existing tools include CS-AMPPred (Porto et al., 2012) (SVM-based classification), PEP-FOLD (Bhadra et al., 2018) (random forest models), Ensemble-AMPPred (Lertampaiporn et al., 2021), and AMPpred-EL (Lv et al., 2022) (ensemble learning approaches). Recently, deep learning-based models such as AMPScanner (Veltri et al., 2018), BERT-AMP (Ma et al., 2022), sAMPpred-GAT (Yan et al., 2022), and AMPpred-MFA (Li et al., 2023) have demonstrated excellent performance in AMP identification tasks. However, most of these approaches suffer from several limitations, including data scarcity, sequence length constraints, limited semantic representation capability, and task specificity. Notably, they generally support only single-task classification and lack a unified framework for predicting multiple functional peptide types such as ACPs and AMPs. Recently, AI (Abbas et al., 2025b) applicable to intelligent healthcare have also begun to emerge. These models are task-specific, designed to fulfill well-defined functions, yet they can only handle the designated task and are unable to adapt to different types of problems.

To overcome these limitations and further enhance the accuracy of ORF prediction and functional peptide identification, a novel deep learning-based gene prediction and functional peptide identification method, namely GP2FI, is proposed. It consists of two components: a gene prediction model, MHA-preconv, and a peptide prediction model, FuncPred-CB. MHA-preconv first extracts candidate ORFs from raw genomic sequences and encodes them with a set of features. It then adopts a two-stage deep learning architecture to integrate both local and global sequence features. FuncPred-CB is a peptide prediction model, which integrates a pre-trained BERT language model with a dual-channel CNN–BiLSTM architecture to effectively handle longer sequences and reduce reliance on manual feature engineering. It is capable of simultaneously predicting ACPs and AMPs within a single unified framework, significantly enhancing the adaptability and accuracy of functional peptide recognition across diverse tasks.

The performance of the MHA-preconv model was evaluated against three widely used tools across multiple genomic datasets. MHA-preconv achieved a gene prediction accuracy of 0.98, outperforming Prodigal (Hyatt et al., 2010) by 0.02, Orphelia (Hoff et al., 2009) by 0.13, and FragGeneScan (Rho et al., 2010) by 0.15, Tiberius (Gabriel et al., 2024) by 0.07, and Helixer (Holst et al., 2025) by 0.08. The FuncPred-CB model was applied to multiple ACP and AMP datasets and conducted systematic comparisons with state-of-the-art prediction methods. FuncPred-CB achieved a maximum accuracy of 0.93 in ACP prediction, exceeding that of ACP-DRL (Xu et al., 2024) by 0.02, ACP-check (Zhu et al., 2022) by 0.15, iACP-DRLF (Lv et al., 2021) by 0.12, and AntiCP2.0 (Agrawal et al., 2020) by 0.22. FuncPred-CB also achieved a competitive accuracy of 0.96 and the highest AUC of 0.99 in AMP prediction, with its accuracy surpassing the deep stacked model AMPpred-MFA (Li et al., 2023) by 0.0113. These experimental results demonstrate the superior performance of GP2FI. MHA-preconv achieves higher accuracy and enhanced sequence modeling capability in gene prediction, while FuncPred-CB balances precision and task generalization in unified ACP and AMP identification, providing a powerful computational foundation for functional peptide discovery and downstream bio-activity research.

## 2 Methods

**Figure 1:**
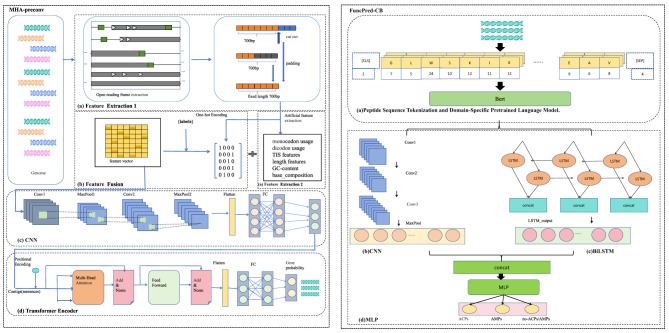
Architecture of the GP2FI framework. **(1)** MHA-preconv model: (a) Feature extraction, (b) Feature fusion, (c) CNN model, and (d) Transformer encoder model. **(2)** FuncPred-CB model: (a) Peptide sequence tokenization and domain-specific pretrained language model, (b) CNN model, (c) Bi-LSTM model, and (d) MLP model.

GP2FI is a two-stage deep learning framework for gene prediction and functional peptide identification. It consists of two stages: a gene prediction model (MHA-preconv) and a functional peptide identification model (FuncPred-CB). MHA-preconv integrates CNNs with Transformer encoder layers to form a two-stage deep architecture, effectively capturing both local sequence patterns and long-range dependencies within ORF sequences. Based on the coding regions identified by MHA-preconv, FuncPred-CB incorporates a pre-trained BERT language model to automatically extract contextual semantic features from amino acid sequences. It also adopts a dual-channel feature extraction mechanism combining CNN and Bi-LSTM, enabling it to simultaneously capture local structural features and global dependencies. Final classification is performed using a multi-layer perceptron.The overall workflow of GP2FI is illustrated in Figure 1.

MHA-preconv comprises four main stages: *feature extraction, multi-feature fusion, CNN model*, and *Transformer encoder model*. First, all candidate ORFs are extracted from the genomic sequence and encoded using one-hot encoding, with each sequence standardized to a fixed length of 700 base pairs. In parallel, six handcrafted features are computed. Then the one-hot encoded sequences and handcrafted features are jointly input into the CNN module to extract local feature patterns. The resulting feature maps are flattened and passed into a Transformer encoder to capture long-range dependencies and global contextual relationships. Finally, a fully connected layer followed by a softmax layer outputs the probability of each ORF being a protein-coding region.

FuncPred-CB also consists of four main stages: *Peptide sequence tokenization and domain-specific pretrained language model, CNN model, Bi-LSTM model*, and *MLP model*. First, the protein sequences translated from MHA-preconv are tokenized, where each amino acid is mapped to a corresponding token ID. The tokenized sequence is then fed into a pretrained BERT language model to obtain contextual semantic embeddings for each residue. The output of BERT is subsequently passed through two parallel channels: a CNN channel for capturing local structural patterns, and a Bi-LSTM channel for modeling long-range dependencies within the sequence.The outputs from both channels are concatenated along the feature dimension and fed into a multi-layer perceptron (MLP) for classification, enabling unified prediction of both ACPs and AMPs.

### 2.1 Feature extraction

**Figure 2:**
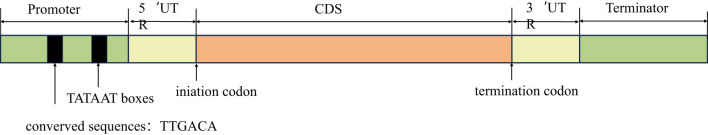
Structure of a prokaryotic open reading frame (ORF).

Each ORF is characterized by six types of effective features, as detailed in Supplementary File S1.1, including *monocodon usage, dicodon usage, translation initiation site (TIS), ORF length, GC content*, and *basic nucleotide composition*. These features are designed to enhance the discriminative power of the model in identifying protein-coding regions. In general, ORFs can be categorized as either complete or incomplete. A complete ORF is defined as one that contains both a start codon (ATG, CTG, GTG, or TTG) and a stop codon (TAG, TGA, or TAA). In contrast, incomplete ORFs lack upstream or downstream regions, or both. In cases where both ends are truncated, the ORF spans the entire sequence fragment without any identifiable start or stop codon. A complete prokaryotic gene, as illustrated in Figure 2, typically begins at the 5′ promoter region and ends at the 3′ terminator region. Transcription occurs between the transcription start site and the transcription termination site, encompassing the 5′ untranslated region (5′ UTR), the ORF, and the 3′ untranslated region (3′ UTR), with only the ORF being translated into protein. Given that the translation initiation site can be located up to 30 base pairs upstream of the canonical start codon, the ORF start offset was set to 30 bp in the search procedure (Hoff et al., 2008). During both training and testing phases, only ORFs with a minimum length of 60 base pairs were considered to ensure reliability.

### 2.2 Multi-feature fusion

The fixed-length ORFs are subjected to one-hot encoding, where each nucleotide is represented by a one-hot vector, resulting each ORF with a length of *L* is represented as an *L*×4 matrix. The encoded ORF and the manually extracted six features are then fused as input for further processing by the subsequent CNN and Transformer encoder layers. The entire feature set, encompassing the encoded ORF, and the six features, is concatenated into a one-dimensional feature vector to represent the input sequence fragment, expressed as:


$$X=\left[X_{ORF},X_{MC},X_{DC},X_{TIS},X_{ORF_{L}},X_{GC},X_{baseC}\right]$$

where *X*_*ORF*_, *X*_*MC*_, *X*_*DC*_, *X*_*TIS*_, *X*_*OR*_*F*__*L*__, *X*_*GC*_, and *X*_*baseC*_ represent the feature extraction vectors mentioned above.

### 2.3 Feature extraction for gene detection

#### 2.3.1 CNN model

A CNN model pre-trained on 10 mutually exclusive datasets, each constructed based on predefined GC content ranges, was employed. The concatenated one-dimensional array obtained from multi-feature fusion is input into the appropriate pre-trained CNN model, which is then fine-tuned using our target dataset. The final CNN architecture consists of six layers. The first layer is a convolutional layer with 64 filters and a filter window size of 3. The second layer is a max-pooling layer with a pool size of 2. The third layer is another convolutional layer with 200 filters and a filter window size of 3. The fourth layer is a second max-pooling layer with a pool size of 2. This is followed by a dropout layer to mitigate overfitting. The output from the convolutional layers is then flattened into a one-dimensional vector and is fed into the first fully connected layer, where the dimensionality is reduced from 35,000 to 4,096.

#### 2.3.2 Transformer encoder model

To incorporate global contextual information, a Transformer encoder modeL was incorporated after the CNN model, allowing long-distance relationships within the sequence be considered while preserving sequential information. The output from the CNN layers is fed into the Transformer encoder with an 8-head attention mechanism to extract global contextual dependencies across the entire ORF sequence. The output is then passed through a flattening layer to convert the multi-dimensional attention output into a one-dimensional vector. This vector is subsequently fed into a fully connected layer with an input dimension of 4,096 and an output dimension of 128, followed by a dropout layer with a dropout rate of 0.2. The resulting vector is then passed into another fully connected layer with 128 neurons, producing a single scalar output. A Sigmoid activation function is applied to obtain the probability that an ORF encodes a protein-coding gene. As a post-processing step, a greedy algorithm (Zhou and Troyanskaya, 2015) is applied to ensure that only one gene is retained among overlapping predictions. The candidate ORF with the highest probability score is selected, and any other ORF overlapping more than 60 base pairs with it is discarded. The final set of predicted genes is then produced.

During model training, the binary cross-entropy loss (Hoff et al., 2008) was used to compute the error between predicted probabilities and ground-truth labels. The model was trained with a batch size of 32 using the Adam optimizer, with a learning rate of 0.001. Multiple hyperparameter configurations were explored to optimize performance.

### 2.4 Peptide sequence tokenization and domain-specific pre-trained language model

The FuncPred-CB model for functional peptide identification begins by tokenizing peptide sequences, converting each amino acid into its corresponding numerical ID as input to the language model. The vocabulary comprises 26 tokens, including the single-letter codes of the 20 standard amino acids, an unknown residue represented by X, and special tokens such as [CLS] and [SEP]. Subsequently, a BERT-based protein language model pre-trained in the ACP-DRL framework is employed to map each amino acid to a vector representation enriched with contextual semantics. Trained on large-scale protein sequence databases using a masked language modeling (MLM) strategy, this model demonstrates strong capabilities in biological sequence modeling and semantic representation.By incorporating BERT-derived contextual embeddings, the model is better equipped to capture critical patterns and long-range dependencies within functional peptides, thereby enhancing the performance of downstream classification tasks.

### 2.5 Feature extraction for functional peptide identification

#### 2.5.1 CNN model

A dual-channel feature extraction architecture based on the pre-trained language model BERT is employed. The sequence representations output by BERT are embedded as high-dimensional, context-sensitive vectors and fed into one of the CNN channels to extract both local structural features and global dependencies. In the CNN channel, the BERT output is transposed to match the input format required by one-dimensional convolution, and then passed through three consecutive convolutional layers. Each layer uses a kernel size of 3 and contains 256, 128, and 64 filters, respectively. After ReLU activation, average pooling is applied to extract stable local pattern features.

#### 2.5.2 Bi-LSTM model

The other feature extraction channel, Bi-LSTM, processes the original sequence outputs from BERT to capture long-range dependencies within the sequence through a Bi-LSTM network. The final hidden state at the last time step is taken as the global representation of the peptide sequence.The features obtained from both CNN and Bi-LSTM channels are concatenated and passed through a batch normalization layer before being fed into a three-layer MLP for classification. This architecture effectively integrates the strengths of CNNs in capturing local structural features with the contextual modeling capabilities of Bi-LSTM, thereby enhancing the overall performance of functional peptide identification. Besides, we further conducted statistical and visual analyses of amino acid composition to explore underlying data characteristics and enhance model interpretability. Specifically, we analyzed the frequency distribution of the 20 standard amino acids in the ACP and AMP datasets. Detailed analysis is provided in Supplementary File S3.2.

During the training phase, binary cross-entropy loss was used as the objective function. The model was optimized using the Adafactor optimizer with an initial learning rate of 2 × 10^−5^. Training was conducted for 20 epochs with a batch size of 4. Early stopping was applied to prevent overfitting, and evaluation metrics including accuracy, F1 score, and Matthews correlation coefficient (MCC) were recorded after each epoch. Comprehensive evaluation metrics are provided in Supplementary File S2.

## 3 Results

### 3.1 Datasets

MHA-preconv was trained and evaluated using four datasets. Dataset_1 contains 164 complete genomes (including bacteria and archaea) and is used for training and validation, with the data split into training and testing sets at a 7:3 ratio. Dataset_2 consists of 10 complete genomes for model tuning. Dataset_3 includes complete genomes from 9 independent species and is used for independent testing. Dataset_4 encompasses 100 newly collected genomes covering broad taxonomic diversity (including Gram-negative bacteria, Staphylococcus spp., etc.) and was divided into five equal subsets for incremental testing. Stratified sampling was applied to both training and test splits to ensure equal expected numbers of positive and negative instances in every mini-batch, preventing the majority class (NCS) from overwhelming the minority class (CDS). In addition, a weighted random-sampling strategy was employed during training to oversample the minority class and further alleviate class bias. All genomic sequences and annotations were downloaded from GenBank (https://www.ncbi.nlm.nih.gov/genbank/) and NCBI RefSeq (https://www.ncbi.nlm.nih.gov/refseq/). The databases used and detailed dataset-construction procedures are described in Supplementary File S3.1.

FuncPred-CB was trained and evaluated by two task-specific datasets For the ACP task, Dataset_1 contains 970 ACPs and 970 non-ACPs, while Dataset_2 includes 861 ACPs and an equal number of non-ACPs.Positive sequences were collected from the AMP and CancerPPD databases and experimentally verified to possess anticancer activity; negatives consist of AMPs without anticancer activity and random peptides extracted from Swiss-Prot. For the AMP task, the dataset contains 10,322 non-redundant AMP sequences whose antimicrobial activity has been experimentally validated, together with 3,029,894 non-AMP sequences. To avoid potential bias, any peptides known to exhibit anticancer activity were excluded from the AMP-positive class, ensuring that positives possess only antimicrobial activity. To mitigate distribution shifts that could arise from random splitting, stratified sampling was applied and a fixed random seed (seed = 702) was set to guarantee reproducibility. Training and test sets are stored in separate physical files to prevent data leakage at the source; the test set is used exclusively for final evaluation and is never involved in model development or hyper-parameter tuning. Class imbalance is addressed by weighted random oversampling of the minority class during training. All datasets were randomly split into training and test sets at an 8:2 ratio. The databases used and detailed dataset-construction procedures are described in Supplementary File S3.1.

### 3.2 Performance of MHA-preconv on the gene dataset Dataset_1 and of FuncPred-CB on the ACP dataset Dataset_1 and the AMP dataset Dataset_3.

**Figure 3:**
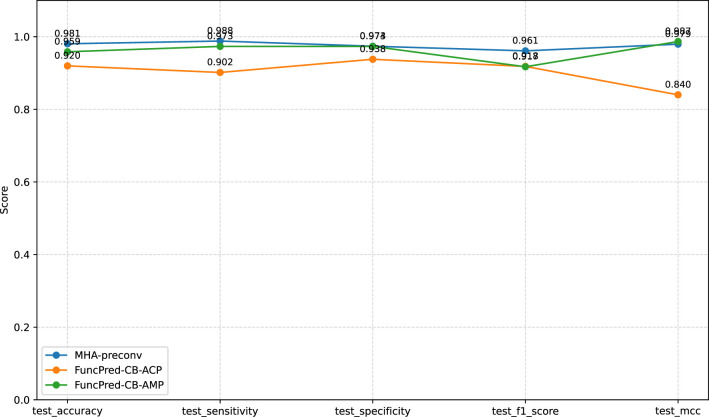
Comparative performance metrics of MHA-preconv and FuncPred-CB on test datasets.

Comprehensive training and testing were performed for gene finding, ACP and AMP tasks with three dedicated models: MHA-preconv, FuncPred-CB-ACP and FuncPred-CB-AMP. MHA-preconv was evaluated on the gene-prediction portion of Dataset_1, whereas FuncPred-CB was assessed on the functional-peptide portions—ACP Dataset_1 and AMP Dataset_3. After 48 epochs, MHA-preconv achieved 98 % test accuracy; FuncPred-CB reached 92 % and 96 % test accuracy on the ACP and AMP datasets, respectively, within 10 epochs. The results are displayed in Figure 3.

### 3.3 Comparison of MHA-preconv with four benchmark methods on Dataset_3 and Dataset_4

MHA-preconv model was compared with five well-established gene prediction tools: Prodigal, Orphelia, FragGeneScan, Tiberius, and Helixer, using Dataset_3 and Dataset_4. The results are shown in Tables 1, 2. The highest accuracy was achieved by MHA-preconv on 8 out of 9 species in Dataset_3. The only exception was N. pharaonis (Species No. 7), where Prodigal slightly outperformed our method. For the remaining species, Acc values of 97.56%, 96.18%, 93.96%, 97.49%, 76.47%, 96.52%, 96.12%, and 96.67% were achieved by MHA-preconv, respectively. On Dataset4, MHA-preconv outperformed all benchmark methods, achieving the highest Acc values across all five subsets: 97.33%, 96.40%, 97.78%, 95.88%, and 95.74%. These demonstrates the strong generalization ability and classification performance of our method across a wide range of microbial genomes.

**Table 1:** Results obtained by four methods on 9 different strains.

**Method**	**Metric**	**1**	**2**	**3**	**4**	**5**	**6**	**7**	**8**	**9**
MHA-preconv	Accuracy(%)	**97.56**	**96.18**	93.96	**97.49**	**76.47**	**96.52**	85.53	**96.12**	**96.67**
	Sn (%)	94.25	**96.36**	92.07	96.43	62.73	**97.12**	82.46	96.11	**96.43**
	Sp (%)	**97.13**	95.15	**93.74**	**97.67**	**84.63**	**95.80**	83.77	**97.38**	**97.53**
	HM (%)	**95.78**	**93.67**	**92.90**	**97.55**	71.97	**96.45**	84.99	94.47	**96.70**
	Precision (%)	**98.54**	90.64	94.71	**97.40**	**90.48**	**95.65**	85.42	95.20	**95.64**
	F1 Score (%)	96.20	**92.94**	93.37	**97.39**	**74.79**	**96.34**	85.46	96.62	**96.62**
Prodigal	Accuracy (%)	95.96	95.40	93.96	95.81	65.15	71.85	91.50	91.17	94.66
	Sn (%)	96.13	96.31	95.77	96.97	64.99	92.27	88.99	97.88	91.96
	Sp (%)	95.97	90.26	93.62	94.81	65.31	81.52	94.17	96.47	97.52
	HM (%)	95.93	92.87	92.82	94.77	65.25	86.56	91.61	97.40	93.68
	Precision (%)	96.70	93.38	96.15	95.07	65.07	89.35	89.90	97.53	93.30
	F1 score (%)	96.32	92.60	96.03	96.93	65.03	90.78	89.06	97.70	92.62
Orphelia	Accuracy (%)	84.36	92.77	72.17	93.36	76.79	71.85	85.37	84.99	89.06
	Sn (%)	80.58	90.46	68.74	89.40	74.23	66.59	76.47	83.79	84.79
	Sp (%)	88.57	95.20	75.99	95.54	79.52	77.51	74.28	86.24	86.24
	HM (%)	83.30	92.70	72.39	93.03	75.51	71.85	85.70	84.97	84.97
	Precision (%)	85.53	91.61	70.43	90.87	75.50	69.13	80.62	85.18	85.18
	F1 score (%)	83.77	91.03	69.57	90.13	74.86	67.83	78.50	84.15	84.15
FragGeneScan	Accuracy (%)	83.57	62.11	72.19	86.19	71.34	57.76	68.19	79.47	59.59
	Sn (%)	82.50	75.86	78.98	84.71	63.22	63.62	74.93	87.54	63.46
	Sp (%)	83.62	52.58	66.47	87.77	77.68	52.89	62.57	72.76	63.67
	HM (%)	83.06	62.24	72.20	86.24	72.30	57.66	68.80	79.44	59.90
	Precision (%)	86.41	68.28	75.75	89.10	77.63	60.66	71.34	83.40	61.34
	F1 score (%)	83.57	71.93	77.13	90.79	72.74	62.06	72.86	85.84	62.23
Tiberius	Accuracy (%)	90.26	93.73	74.33	93.00	75.75	72.34	87.21	86.94	89.50
	Sn (%)	83.82	90.46	65.27	89.80	74.76	68.49	80.33	83.90	84.67
	Sp (%)	89.51	95.20	73.30	96.72	78.23	79.54	73.26	87.15	88.45
	HM (%)	84.03	91.36	72.92	93.22	76.12	71.57	85.37	83.90	85.39
	Precision (%)	85.53	89.11	72.34	91.88	74.90	70.40	81.34	85.89	86.17
	F1 score (%)	83.77	90.32	69.99	90.41	75.45	68.82	79.49	85.13	85.87
Helixer	Accuracy(%)	89.42	90.46	76.65	93.06	77.10	70.21	84.69	86.44	82.09
	Sn (%)	79.58	76.89	72.31	89.71	74.66	65.02	79.08	80.72	78.41
	Sp (%)	84.16	88.27	68.24	79.27	78.90	72.33	86.61	85.60	87.58
	HM (%)	83.00	80.44	71.14	79.20	74.33	66.45	85.37	80.11	79.95
	Precision (%)	83.79	84.00	79.52	86.52	74.85	68.96	81.34	85.49	85.83
	F1 score (%)	83.60	82.03	72.36	87.40	75.60	66.63	79.49	84.22	79.40

The bold values indicate the best (optimal) results, which correspond to the highest performance achieved for the respective metrics.

**Table 2:** Results obtained by the four methods on the five divided subsets.

**Method**	**Metric**	**A**	**B**	**C**	**D**	**E**
MHA-preconv	Accuracy (%)	**97.33**	**96.40**	**97.78**	**95.88**	**95.74**
	Sn (%)	**92.13**	**93.90**	81.89	**90.75**	92.13
	Sp (%)	**98.43**	**97.65**	**99.71**	**97.55**	**97.55**
	HM (%)	**95.56**	**95.74**	**89.92**	**94.03**	94.76
	Precision (%)	**99.28**	**95.21**	**99.29**	**94.86**	**94.93**
	F1 score (%)	89.75	94.55	**89.75**	**92.76**	93.51
Prodigal	Accuracy (%)	95.30	94.60	84.56	85.37	94.71
	Sn (%)	92.10	90.00	85.04	72.21	95.66
	Sp (%)	90.02	89.00	78.09	70.23	90.18
	HM (%)	95.45	93.01	81.17	74.56	97.01
	Precision (%)	88.20	87.03	80.01	70.73	93.82
	F1 score (%)	95.49	93.48	81.52	74.28	97.52
Orphelia	Accuracy (%)	83.96	92.96	73.12	76.40	84.67
	Sn (%)	84.55	92.02	74.02	78.25	85.09
	Sp (%)	82.36	91.20	70.40	75.44	82.65
	HM (%)	88.03	95.08	76.64	80.02	86.86
	Precision (%)	78.87	89.80	66.71	72.99	81.22
	F1 score (%)	87.57	95.20	74.95	79.06	86.25
FragGeneScan	Accuracy (%)	81.89	72.30	84.21	71.50	59.13
	Sn (%)	84.01	76.85	85.30	70.71	58.02
	Sp (%)	80.12	68.02	85.24	68.34	55.36
	HM (%)	75.54	67.50	81.11	70.99	58.27
	Precision (%)	78.02	66.82	83.67	67.23	54.73
	F1 Score (%)	75.23	66.45	80.92	71.02	56.28
Tiberius	Accuracy (%)	82.85	79.64	84.56	74.60	78.61
	Sn (%)	76.21	77.44	85.63	76.81	69.25
	Sp (%)	86.32	65.65	80.24	65.30	75.06
	HM (%)	74.55	70.05	82.44	72.02	71.23
	Precision (%)	83.92	79.86	85.00	74.01	74.33
	F1 Score (%)	78.20	77.00	85.92	74.90	76.29
Helixer	Accuracy (%)	82.43	76.90	82.11	74.99	79.10
	Sn (%)	75.41	67.95	74.05	69.91	66.88
	Sp (%)	88.47	79.63	79.27	78.54	80.30
	HM (%)	75.86	70.00	79.14	70.21	63.90
	Precision (%)	79.12	74.15	83.91	76.11	77.70
	F1 Score (%)	77.03	70.60	82.10	71.66	70.21

The bold values indicate the best (optimal) results, which correspond to the highest performance achieved for the respective metrics.

### 3.4 Comparison with state-of-the-art ACP methods on Dataset_1, and Dataset_2

**Figure 4:**
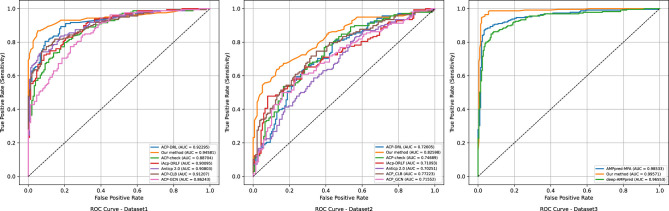
Comparison of ROC curves for different models across three functional peptide datasets.

To comprehensively evaluate the proposed FuncPred-CB model for functional-peptide recognition, we conducted comparative experiments on two benchmark datasets against six state-of-the-art ACP predictors: ACP-DRL, ACP-check, iACP-DRLF, AntiCP 2.0, ACP-CLB (Geng et al., 2025) and ACP-GCN (Rao et al., 2020). As illustrated in Figure 4 and Table 3, FuncPred-CB achieves 92.49% accuracy, 91.19% sensitivity, 93.78% specificity, 86.78% MCC and 94.58% AUC on Dataset_1, and 73.19% accuracy, 71.01% sensitivity, 77.20% specificity, 46.42% MCC and 82.60% AUC on Dataset_2. Except for a marginally lower accuracy than ACP-CLB, FuncPred-CB surpasses all baseline methods on the remaining metrics, demonstrating robust adaptability to complex and heterogeneous data. Collectively, these results validate the superior overall performance, generalizability and competitiveness of FuncPred-CB as a functional-peptide predictor for anticancer-peptide identification.

**Table 3:** Performance comparison of various models on the Datasets_1 and Datasets_2.

**Model**	**Dataset**	**Acc (%)**	**Sn (%)**	**Sp (%)**	**MCC (%)**	**AUC (%)**
ACP-DRL	Dataset_1	90.67	86.01	**95.33**	80.80	92.30
	Dataset_2	67.39	69.57	69.57	34.80	72.74
FuncPred-CB	Dataset_1	92.49	**91.19**	93.78	86.78	**94.58**
	Dataset_2	73.19	**71.01**	**77.20**	46.42	**82.60**
ACP-check	Dataset_1	90.24	90.52	90.52	86.45	88.70
	Dataset_2	65.58	70.29	72.31	31.30	74.69
iACP-DRLF	Dataset_1	80.65	80.22	85.51	61.26	90.10
	Dataset_2	62.32	59.42	63.42	24.68	71.10
AntiCP 2.0	Dataset_1	70.97	76.12	67.92	42.14	90.80
	Dataset_2	58.70	65.94	60.57	17.58	70.25
ACP-CLB	Dataset_1	**94.33**	90.12	93.24	**87.50**	91.21
	Dataset_2	**79.10**	69.35	74.40	**60.61**	77.22
ACP-GCN	Dataset_1	84.12	80.17	84.69	64.83	86.24
	Dataset_2	70.90	66.95	70.00	39.70	71.55

The bold values indicate the best (optimal) results, which correspond to the highest performance achieved for the respective metrics.

### 3.5 Comparison with the latest AMP method on Dataset_3

To assess the performance of FuncPred-CB on AMP recognition, we benchmarked it against the recent models AMPpred-MFA and deep-AMPpred (Zhao et al., 2025) on Dataset_3. The results, provided in Supplementary File S3.3 and Table 4, show that FuncPred-CB attained 95.9 % accuracy, 97.3 % sensitivity, 96.3 % specificity, 91.7 % MCC, and 98.7 % AUC. Although its accuracy is marginally lower than that of deep-AMPpred, FuncPred-CB delivers superior overall performance relative to both AMPpred-MFA and deep-AMPpred. These findings underscore that FuncPred-CB not only excels in anticancer-peptide prediction but also exhibits strong discriminative power and generalizability in AMP classification tasks.

**Table 4:** Comparison of performance metrics between FuncPred-CB, AMPpred-MFA and Deep-AMPpred on the Dataset_3.

**Method**	**Acc (%)**	**Sn (%)**	**Sp (%)**	**MCC (%)**	**AUC (%)**
FuncPred-CB	95.85	**97.33**	**97.30**	**91.72**	**98.67**
AMPpred-MFA	94.72	94.74	94.00	88.00	98.53
Deep-AMPpred	**97.28**	90.70	93.22	80.60	96.55

The bold values indicate the best (optimal) results, which correspond to the highest performance achieved for the respective metrics.

### 3.6 The impact of basic nucleotides on MHA-preconv

Since the differences in base frequencies between coding and non-coding regions can reflect structural and functional characteristics of the genome, the data distribution of coding and non-coding regions was plotted (detailed in Supplementary File S3.4), and the impact of including nucleotide composition as a feature on model performance was compared. As shown in Table 5, the incorporation of nucleotide composition features improved the model's prediction accuracy, sensitivity, specificity, and other metrics across different genomes.

**Table 5:** Comparison of the impact of nucleotide composition features on the performance of MHA-preconv.

**Genome ID**	**base_composition**			**nobase_composition**		
	**Acc (%)**	**Sn (%)**	**Sp (%)**	**Acc (%)**	**Sn (%)**	**Sp (%)**
NC_002516.1	97.33	95.33	98.04	94.26	92.40	94.92
NC_000909.1	97.24	95.16	97.97	95.70	95.50	97.35
NC_007426.1	97.18	94.98	98.04	93.32	92.83	93.49
NC_002528.1	97.30	97.23	95.43	85.79	76.33	85.07
NC_007164.1	97.17	98.02	97.04	96.70	94.56	97.71
NC_002932.1	97.56	96.83	97.24	93.63	95.42	93.00
NC_000921.1	97.30	98.22	96.84	95.32	94.21	97.38
NC_007577.1	97.43	96.43	97.33	88.37	91.96	78.99
NC_006833.1	97.34	95.83	97.24	95.68	95.68	94.67
NC_006350.1	97.29	95.07	98.07	96.35	95.94	96.50

Accordingly, the use of standalone CNN and Transformer modules was also compared, different configurations of CNN and encoder layers were tested, and the effect of the Bi-LSTM model on model performance was evaluated. All these modifications significantly enhanced the model's performance. Detailed analyses and results are provided in Supplementary Files S3.5–S3.8.

### 3.7 Physicochemical property analysis of functional peptides

**Figure 5:**
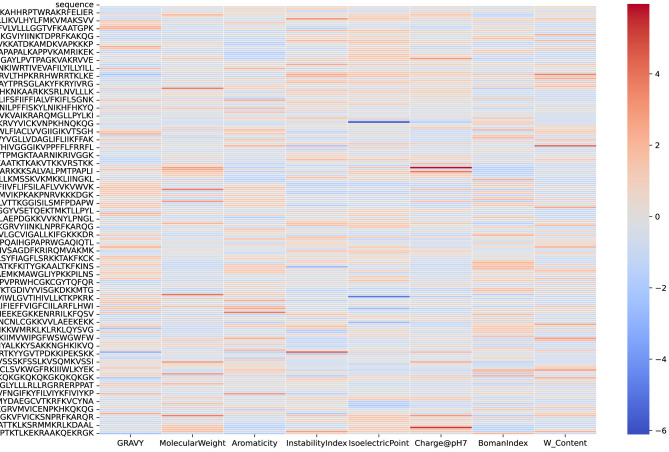
Heatmap of physicochemical property profiles for functional peptides predicted by the GP2FI model.

**Figure 6:**
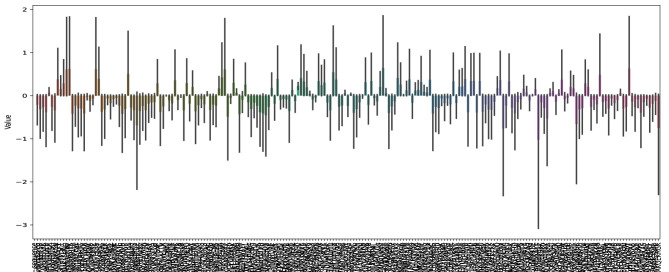
Bar chart of key physicochemical properties for functional peptide sequences predicted by the GP2FI model.

**Figure 7:**
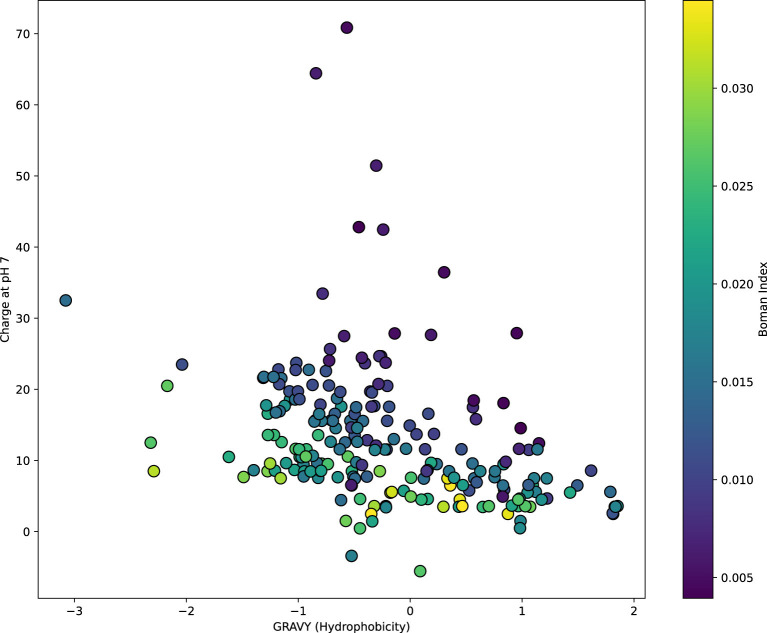
Scatter plot of GRAVY and charge properties for functional peptide sequences predicted by the GP2FI model.

To evaluate the potential of the predicted functional peptides in the fields of ACPs and AMPs, we employed the unified prediction framework GP2FI. Coding genes predicted from Dataset1 were translated into protein sequences, which were then subjected to functional peptide identification using FuncPred-CB and comprehensive multiparametric physiochemical characterization. As shown in Figures 5–7, the large number of sequences limits the resolution of the heat-maps, which are therefore intended only as an overview of the global physiochemical landscape of ACPs and AMPs. Detailed sequence information and peptide classifications, together with *in-silico* mapping against the CancerPPD database, are provided in Appendix S3.10; wet-lab functional assays will be required for definitive validation.

Figure 5 illustrates the normalized distribution of eight physicochemical properties—GRAVY (hydrophobicity), molecular weight, aromaticity, instability index, isoelectric point, net charge at pH 7, Boman index, and tryptophan content (W_Content)—across multiple sequences predicted as functional peptides. The heatmap reveals substantial variation in these properties among different sequences, indicating their potential functional divergence. For example, the sequence in row 9 shows high values for isoelectric point, net charge, and Boman index, suggesting strong cationic affinity and protein interaction capability, making it a promising ACP candidate. Similarly, the sequence in row 19 exhibits elevated isoelectric point and positive charge, along with moderate aromaticity and tryptophan content, indicating considerable anticancer potential. Sequences in rows 13 and 41 also display favorable profiles across several ACP-associated features, highlighting their structural suitability as ACPs. In contrast, the sequence in row 35 exhibits high GRAVY and aromaticity values, reflecting strong hydrophobicity and structural stability—characteristics well-suited for AMP candidates. The sequence in row 16 also scores high in GRAVY and instability index, indicating favorable membrane affinity and antimicrobial stability. Row 43 shows marked enrichment in aromaticity and tryptophan content, aligning with classic physicochemical traits of AMPs.

To more intuitively reveal the numerical differences in key properties and their association with sequence characteristics, Figure 6 presents bar charts of GRAVY, Boman index, and tryptophan content across the predicted functional peptide sequences. Compared to the heatmap, the bar plots offer a clearer depiction of the magnitude of each attribute, facilitating the identification of representative sequences. For instance, these visualizations further confirm that sequences such as that in row 35 exhibit notably high scores in GRAVY and aromaticity, indicating strong hydrophobicity and high structural stability—traits that suggest strong potential as AMP candidates.

Furthermore, Figure 7 presents a scatter plot illustrating the relationship between GRAVY and net charge, with the Boman index encoded as the color gradient. This visualization reveals the multidimensional interaction among these physicochemical properties. Analysis shows that most AMP-like sequences cluster in the region characterized by high GRAVY and low charge, whereas sequences exhibiting moderate hydrophobicity, medium to high charge, and elevated Boman index tend to group in the ACP-favored region. Brighter colors (yellow) represent higher Boman index values, indicating stronger potential for protein–protein interactions and possibly higher biological activity. For instance, sequences located in the upper-right region of the plot exhibit both high charge and high Boman index, suggesting strong structural characteristics associated with ACPs, while those in the lower-left region with low charge and high hydrophobicity are more typical of AMP-like features.

## 4 Discussion

The main innovations of GP2FI are reflected in the following aspects: First, to address the challenges of gene prediction, the MHA-preconv model integrated CNN and Transformer architectures, enabling the effective extraction of local patterns in ORFs while simultaneously capturing long-range dependencies in nucleotide sequences. Compared with previous models that heavily rely on handcrafted features, a more streamlined architecture, higher prediction efficiency, and reduced dependency on manual feature inputs are achieved by MHA-preconv. Second, the FuncPred-CB model was proposed for dual-task functional peptide prediction. It leverages a pretrained BERT language model to automatically extract contextual semantic representations of amino acid sequences and employs a dual-channel architecture combining CNN and Bi-LSTM to deeply fuse local and global features. Experimental results demonstrate that superior performance across multiple metrics is achieved by FuncPred-CB. Finally, a physicochemical property analysis of the predicted functional peptide sequences was conducted. The results further validated the predictive effectiveness of the model and revealed functional tendencies of different sequences in anticancer and antimicrobial directions, offering valuable insights for subsequent experimental validation and drug development.

Despite its strong predictive performance, GP2FI has several limitations. First, the framework only provides “precursor-level” activity scores for small ribosomally encoded peptides (sREPs) translated via the canonical ribosome; it ingests complete metagenomic ORF sequences and currently does not model signal-peptide removal, proteolytic cleavage, post-translational modifications, or non-ribosomal peptide synthetase (NRPS) pathways. Dedicated maturation modules and wet-lab validation will be incorporated in future work to bridge this gap. In addition, the pipeline remains a two-stage system that requires manual hand-off between gene prediction and peptide function prediction. We plan to introduce joint training to establish a truly end-to-end workflow. The current model also exhibits suboptimal efficiency when handling very long sequences and in deployment scenarios, while physiochemical property analysis is not yet embedded within the predictive loop. Upcoming efforts will focus on integrating structural information to build more biologically interpretable models with enhanced explainability.We acknowledge that, although our study has significantly reduced reliance on manual features compared with traditional approaches, a subset of handcrafted prior features is still employed. In future work, we are committed to achieving high-efficiency prediction without any handcrafted features whatsoever.

Certainly, as Mc Neil and Lee (2025) emphasized, expanding and prospecting future functional-peptide research requires “novel immunomodulatory molecules to overcome resistance”—a niche for which ACPs are ideal candidates. Rapid and accurate genome-wide identification of ACPs equips clinicians with a readily deployable “peptide arsenal” that can be immediately combined with ICIs, perfectly aligning with the review's vision of “personalized ICI-combination therapy.” In our future work, we plan to adopt the multi-model voting ensemble framework proposed by Abbas et al. (2025c), originally designed for multi-class peptide tasks, to further boost the robustness and generalizability of our prediction system.

## 5 Conclusion

In this study, a unified prediction framework, GP2FI, was proposed, which integrates two deep learning models: MHA-preconv for metagenomic gene prediction and FuncPred-CB for the identification of ACPs and AMPs. As a multitask integrated deep learning framework, GP2FI exhibits excellent performance and practical application potential in both gene prediction and functional peptide recognition. Compared to traditional methods, experimental results across multiple datasets demonstrate that strong performance advantages and broad adaptability in both coding gene detection and functional peptide screening are offered by GP2FI. Ongoing improvements in data integration, model efficiency, and biological interpretability will further strengthen its utility, providing comprehensive computational support for the efficient discovery of functional peptide-based therapeutics.

## Data Availability

Gene prediction-related datasets can be obtained from NCBI RefSeq (https://www.ncbi.nlm.nih.gov/refseq/) and GenBank (https://www.ncbi.nlm.nih.gov/genbank/). The CAMI dataset is available for download at https://data.cami-challenge.org/. The Sharon real metagenomic dataset can be accessed from the NCBI SRA database (https://www.ncbi.nlm.nih.gov/sra). Cancer peptide datasets can be obtained from the following databases: DADP (http://webs.iiitd.edu.in/raghava/dadp/), CAMP (http://www.camp.bicnirrh.res.in/), APD/APD2 (https://aps.unmc.edu/), CancerPPD (http://crdd.osdd.net/raghava/cancerppd/), UniProt (https://www.uniprot.org/), and SwissProt (https://www.uniprot.org/help/swiss-prot). Antimicrobial peptide datasets can be obtained from ADAM (http://bioinform.info/adam/), APD (https://aps.unmc.edu/), CAMP (http://www.camp.bicnirrh.res.in/), and LAMP (http://biotechlab.fudan.edu.cn/database/lamp/).
