# Supplementary material for: Multi-feature fusion for gene prediction and functional peptide identification
Source: Front Microbiol. 2026 Feb 6;17:1736391. doi: 10.3389/fmicb.2026.1736391 (PMC12920568; doi:10.3389/fmicb.2026.1736391)
Supplement: Supplementary file 1 [file Supplementary_file_1.docx]

Multi-Feature Fusion for Gene Prediction and Functional Peptide Identification Multi-Feature Fusion for Gene Prediction and Functional Peptide Identification

Supplementary information

Ma Chenjing

June 18, 2025

**S1 Methods**

**S1.1 Feature Extraction**

**(1) Monocodon Usage**

Since codon usage preferences are specific to different species and even to different genes within the same species, codon usage is a useful feature for distinguishing between coding and non-coding ORFs. Therefore, the monocodon usage frequencies of all possible ORFs are calculated to generate a vector:

$$\begin{matrix} X_{MC}\text{=}\left[f_{1}^{c},...,f_{i}^{c},...,f_{64}^{c}\right] \\ f_{i}^{c}=\frac{n_{i}}{N} \end{matrix}$$

where, represents the frequency of the $i\text{-}th$ codon, denotes the number of occurrences of the $i\text{-}th$ codon, and $N$is the total number of codons in an ORF.

**(2) Dicodon Usage**

The rationale for using dicodon usage frequency as a feature is similar to that of monocodon usage, as codon usage bias is closely associated with gene expression levels. Therefore, dicodon usage is also incorporated as a useful feature for distinguishing between coding and non-coding ORFs. By calculating the dicodon frequency of an ORF, a segment is represented using the following vector :

$$\begin{matrix} X_{DC}\text{=}\left[f_{11}^{c},...,f_{ij}^{c},...,f_{64,64}^{c}\right] \\ f_{ij}^{dc}=\frac{n_{ij}}{N-1} \end{matrix}$$

where represents the frequency of the dicodon pair $\left(i,j\right)$ within an ORF.

**(3) TIS**

The TIS marks the beginning of protein synthesis and is therefore critical for accurately identifying the boundaries of coding regions[1]. TIS prediction is typically expressed as a probabilistic score, indicating the likelihood that a given sequence window contains a TIS. A higher score reflects greater confidence in the presence of a TIS within the ORF. The feature vector can then be represented as:

$$\begin{matrix} F=N\left(E\left(S\right)\right) \\ P=M\left(F\right) \end{matrix}$$

where $S$ represents the sequence window centered around the potential start codon., $E$ denotes the one-hot encoding function, $N$represents the normalization function, and $P$ is the prediction of whether a TIS exists within the sequence window.

**(4) ORF Length**

Research has indicated that the length of an ORF can serve as an indicator of whether a sequence is likely to encode a protein. Longer ORFs are more likely to encode proteins[2], as the length of coding ORFs is significantly greater than that of non-coding ORFs, given that they require longer sequences to encode a complete protein[3]. Consequently, we can represent the metagenomic fragment by calculating the ratio of the ORF length to the fragment length, using the following feature vector $X_{ORFC}$:

$$X_{ORFC}=\frac{l}{L}$$

where $L$ represents the length of the ORF, and $l$ denotes the length of the fragment.

**(5) GC Content**

The GC content typically varies between coding and non-coding regions[4]. The GC content is calculated as the ratio of the number of guanine (G) and cytosine (C) bases to the total number of bases in the sequence. The following vector is used to represent the sequence fragment:

$$\begin{matrix} X_{GC}=\left[gc_{1},gc_{2},...,gc_{n}\right] \\ gc_{i}=\frac{c_{G}+c_{C}}{N}\text{×}100\text{\%} \end{matrix}$$

where $GC\_i$represents the$GC$content percentage of the$i\text{-}th$sequence, $n$signifies the total number of sequences, $G$denotes the content of guanine (G) in the sequence, $\text{C}$indicates the content of cytosine (C) in the sequence, and $GC\_$total is the total GC content in the ORF.

**(6) Nucleotide Composition**

Due to significant differences in base composition among various genes, the content of bases (A, T, C, G), in addition to GC content, can also serve as a feature to distinguish coding regions. The distribution of different base content in coding regions is depicted in Fig. S1. Gene prediction relies not only on GC content but also on the content of other bases. The proportions of adenine, thymine, guanine, and cytosine are represented as $X_{A}$, $X_{T}$, $X_{G}$, and $X_{C}$, respectively:

$X_{A}=\frac{n_{A}}{N}$

$X_{T}=\frac{n_{T}}{N}$

$X_{G}=\frac{n_{G}}{N}$

$X_{C}=\frac{n_{C}}{N}$

$X_{baseC}=\frac{n_{A}+n_{T}+n_{G}+n_{C}}{N}$

$$n_{A}+n_{T}+n_{G}+n_{C}=N$$

where $N$ denotes the total number of bases in the sequence, ,, and represent the counts of adenine (A), thymine (T), guanine (G), and cytosine (C) in the sequence, respectively.


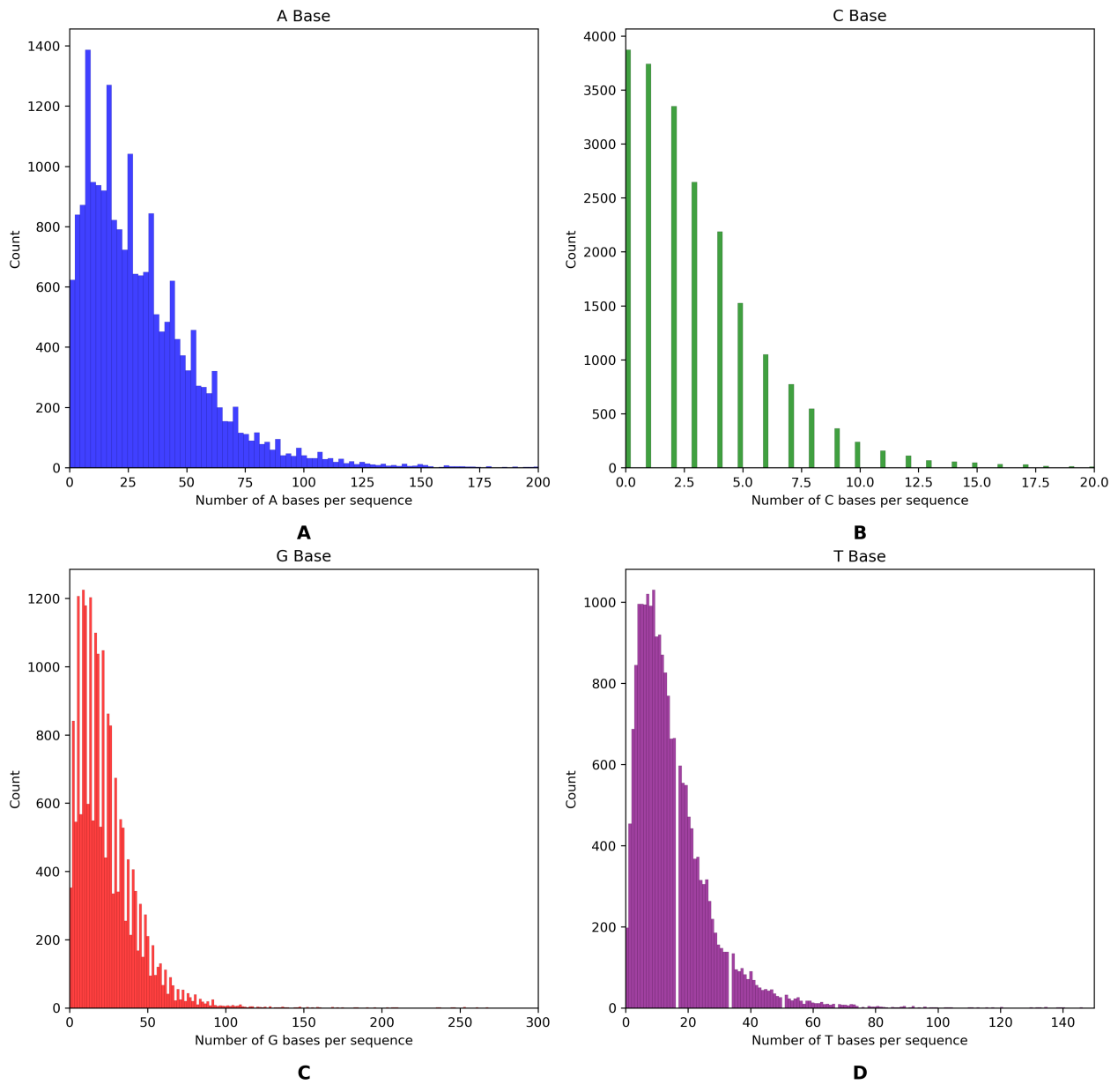


Fig. S1. Distribution of Base Content in Coding Regions.

**S2 Performance Metrics**

In this study, eight key evaluation metrics are evaluated to assess the performance of GP2FI: sensitivity, specificity, harmonic mean, accuracy, Matthews correlation coefficient (MCC), precision, F1 score, and area under the ROC curve (AUC). These metrics are widely used in the evaluation of deep learning models and collectively reflect the accuracy, robustness, and stability of classification performance. The definitions of these metrics are as follows:

$Sensitivity=\frac{TP}{TP+FN}$

$Specificity=\frac{TP}{TP+FP}$

$$Harmonic Mean=\frac{2\times Sensitivity\times Specificity}{Sensitivity+Specificity}$$

$$A\text{cc}uracy=\frac{TP+TN}{TP+TN+FP+FN}$$

$$MCC=\frac{TP\times TN-FP\times FN}{\sqrt{\left(TP+FP\right)\left(TP+TN\right)\left(TN+FP\right)\left(TN+FN\right)}}$$

$$Precision=\frac{TP}{TP+FP}$$

$$F1 score=2\times\frac{Precision\times Recall}{Precision+Recall}$$

$$TPR=\frac{TP}{TP+FN}$$

$$FPR=\frac{FP}{FP+TN}$$

where $TP$represents the number of true positive samples, $FP$represents the number of false positive samples, $TN$represents the number of true negative samples, and $FN$represents the number of false negative samples.

**S3 Results**

**S3.1 Datasets**

**(1) Gene Prediction Datasets**

This study employs four genomic datasets. Dataset_1, Dataset_2 and Dataset_3 were derived from those published in Orphelia[5] and MGC[6]; we have updated their accession numbers and strain annotations to reflect current RefSeq records. Dataset_1 comprises 164 complete genomes—131 fully sequenced bacterial and archaeal isolates together with 33 Gram-negative bacteria randomly selected from NCBI RefSeq[7]—and was split 8:2 into training and validation subsets for MHA-preconv development. Dataset_2 contains ten complete genomes reserved exclusively for debugging and hyper-parameter tuning. Dataset_3 serves as an independent test set of two archaeal and seven bacterial genomes, each providing tens of thousands of annotated genes, and is used to assess generalisation performance. To probe predictive capability across novel taxonomic diversity, we constructed Dataset_4 by extracting un-annotated, high-confidence gene sequences from the CAMI[8] (https://data.cami-challenge.org/ ) and Sharon[9] (https://www.ncbi.nlm.nih.gov/sra ) collections, aligning them against NCBI RefSeq with BLAST[10] and retaining only high-similarity matches; this yielded 100 newly recognised genomes spanning Gram-negative bacteria, Staphylococcus spp., and other lineages. Dataset_4 was randomly divided into five equal subsets (Dataset4_A–E) to facilitate incremental testing. Both training and test sets were subjected to stratified sampling, so that the expected number of positive and negative instances is equal within each mini-batch, preventing the majority class (NCS) from overwhelming the minority class (CDS). Additionally, we implemented a weighted random sampling technique that oversamples the minority class during training, further mitigating the model's bias toward the majority class. All genomic sequences and annotations were downloaded from GenBank[11] (https://www.ncbi.nlm.nih.gov/genbank/ ) and NCBI RefSeq (https://www.ncbi.nlm.nih.gov/refseq/ ). Annotated genes serve as positive instances, while all possible ORFs identified within intergenic regions constitute negative examples. To ensure adequate training and test coverage, we randomly extract 700-bp fragments: 1× genome coverage for each training genome and 5× coverage for each test genome, where n-fold genome coverage is defined as the total length of sampled DNA being n times the complete length of the original genome sequence.

**(2) Functional Peptide Identification Datasets**

To ensure fair comparison, this study employs two standard task-specific datasets: the ACP dataset and the AMP dataset, which are respectively used for training and evaluation of the dual-task model. For the ACP prediction task, data were obtained from Dataset1 and Dataset2 provided by AntiCP 2.0[12], which integrate multiple authoritative sources, including DADP[13], CAMP[14] , APD[15] , APD2[16] , CancerPPD[17] , UniProt[18] , and SwissProt[19] . Dataset1 contains 970 ACPs and 970 non-ACPs, Dataset2 includes 861 ACP sequences and an equal number of non-ACPs. Positive samples were collected from AMP and CancerPPD databases and exhibit experimentally verified anticancer activity. Negative samples consist of AMPs lacking anticancer properties and random peptide segments extracted from SwissProt.

In the AMP prediction task, the dataset used was provided by Ma et al.[20], sourced from four public databases: ADAM[21] , APD[15], CAMP[14] , and LAMP[22] , yielding a total of 10,327 AMP sequences. After removing duplicate entries and sequences longer than 300 amino acids, 10,322 non-redundant AMPs were retained, 55.17% of which are shorter than 50 amino acids. To avoid potential bias, peptides with known anticancer activity were excluded from the AMP-positive class, ensuring that all positive samples represent genuine antimicrobial activity. Negative samples for the AMP task were obtained from the UniProt  database by filtering for cytoplasm-localized proteins, while excluding any entries containing keywords such as “antimicrobial,” “antiviral,” or “antifungal.” After deduplication and removal of sequences identical to any AMP entries, a total of 3,029,894 non-AMP sequences were retained, including 114,995 sequences shorter than 50 amino acids.

To mitigate potential distribution bias introduced by random splitting, this study employs a stratified sampling strategy for dataset partitioning, with a fixed random seed (seed=702) applied to ensure the reproducibility of the division process. The training and test sets are stored in separate physical files to prevent data leakage at the source, and the test set is used exclusively in the final evaluation stage, without any involvement in model development or tuning. To address the issue of class imbalance, a weighted random sampling mechanism is incorporated during training, which involves oversampling the minority class. Finally, all datasets were randomly split into training and testing sets at an 8:2 ratio.

**S3.2 Comparison of Amino Acid Distribution Features**

Although this study adopts a BERT-based pretrained model to automatically extract deep semantic features from peptide sequences, combined with CNN and Bi-LSTM modules for effective feature modeling in functional peptide prediction, we further conducted a statistical and visual analysis of amino acid composition to explore the underlying data characteristics and enhance model interpretability. Specifically, we analyzed the frequency distribution of the 20 standard amino acids in both ACP and AMP datasets, as shown in Fig. S2. Figures **4A** and **4B** illustrate the amino acid composition differences between functional peptides (ACPs) and non-functional peptides (Non-ACPs) in two ACP datasets from different sources, while **Figure 4C** presents a frequency comparison in the AMP dataset. TThe results show that lysine (K) is significantly enriched in ACPs within Dataset1 (Figure 4A), whereas in Dataset2 (Figure 4B), the difference is less pronounced. In the AMP dataset (Figure 4C), K appears more frequently in Non-AMP sequences, indicating an opposite trend. Additionally, leucine (L) and the negatively charged amino acids aspartic acid (D) and glutamic acid (E) are more prevalent in non-functional peptides, suggesting a negative correlation with peptide functional activity. It is noteworthy that although ACPs and AMPs share some common amino acid preferences, they also exhibit distinct compositional characteristics. For instance, the absolute frequencies of several amino acids, such as K and L, are significantly higher in AMP sequences than in ACPs, as shown in **Figure 4C** compared to **Figures 4A and 4B**. This may be attributed to the broader diversity and generally longer sequence lengths of AMP data. Furthermore, AMP sequences exhibit a more balanced distribution between hydrophilic and hydrophobic residues, which may be related to the structural demands of broad-spectrum antimicrobial activity. In summary, this compositional analysis not only reveals fundamental differences between functional and non-functional peptides at the primary sequence level, but also further validates the biological plausibility and sequence learning capability of the proposed **FuncPred-CB** model.


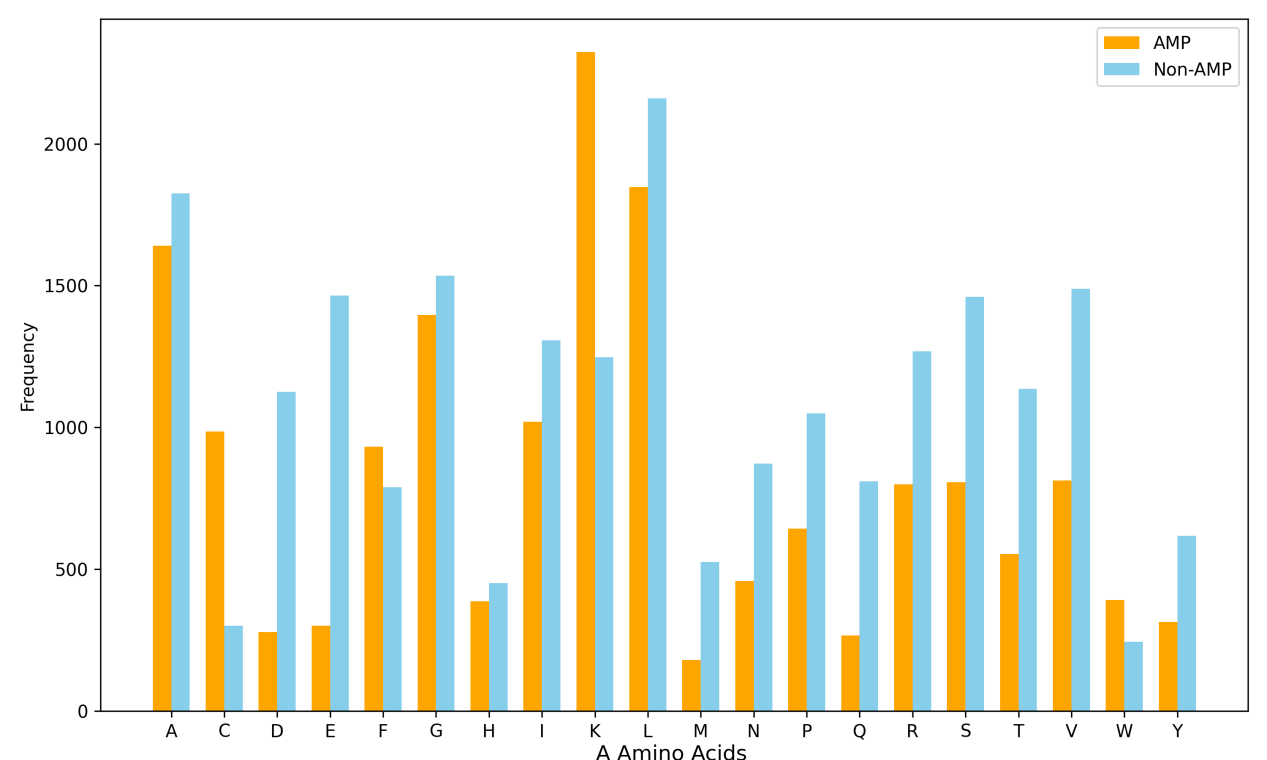


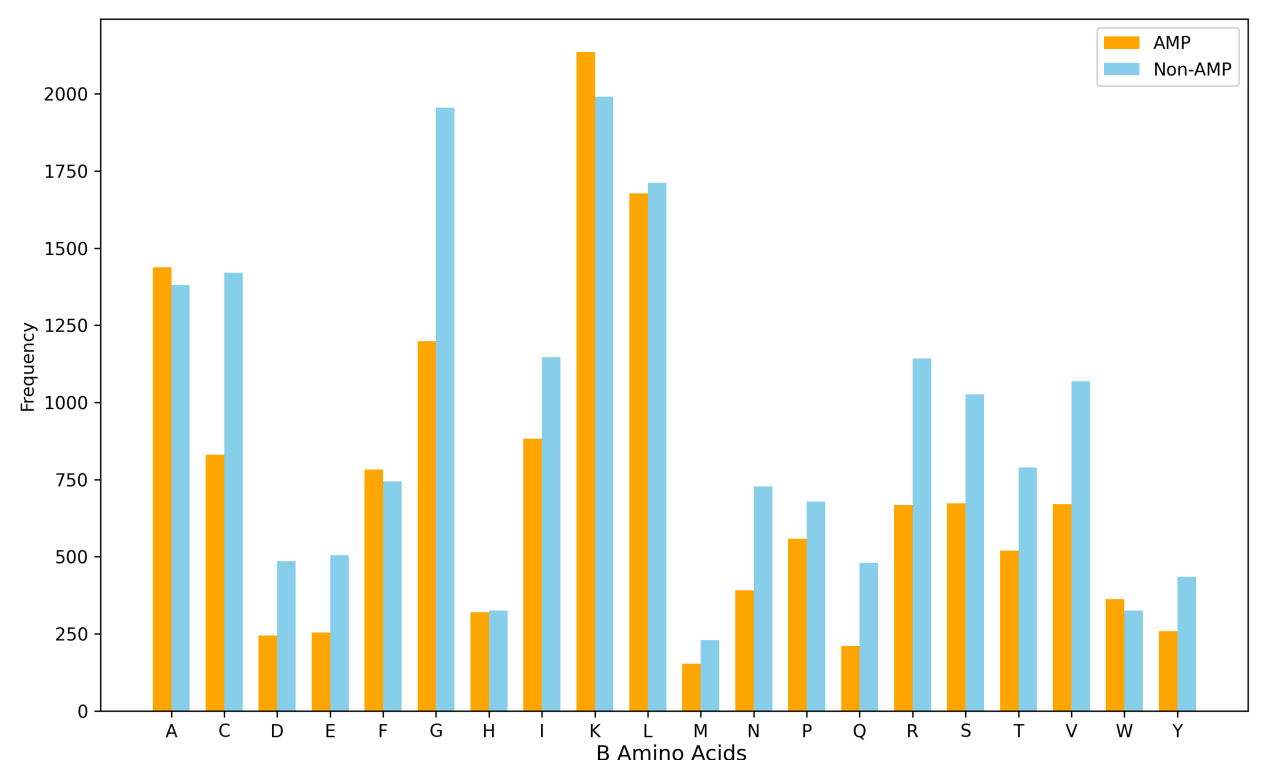


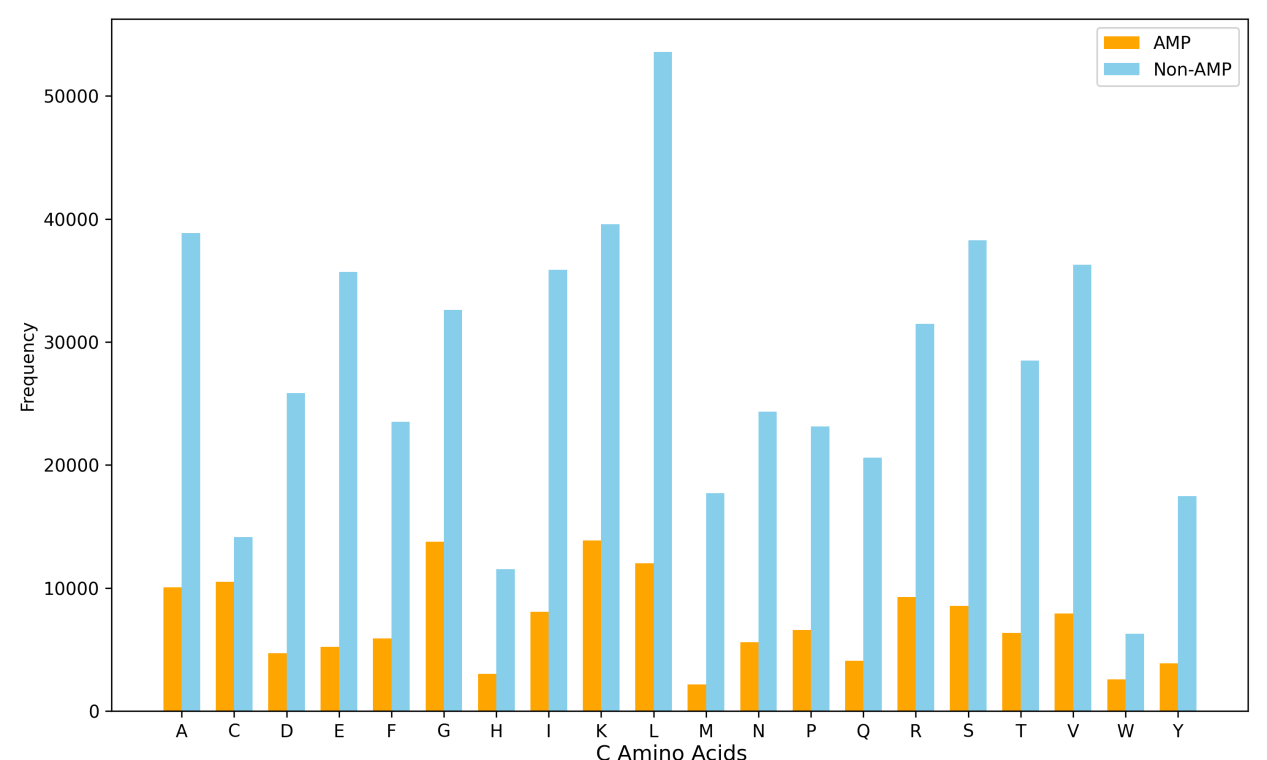


Fig. S2.Amino acid frequency distribution of ACPs and Non-ACPs in functional peptide datasets.

**S3.3 Comparison with the Latest AMP Method on Dataset_3**


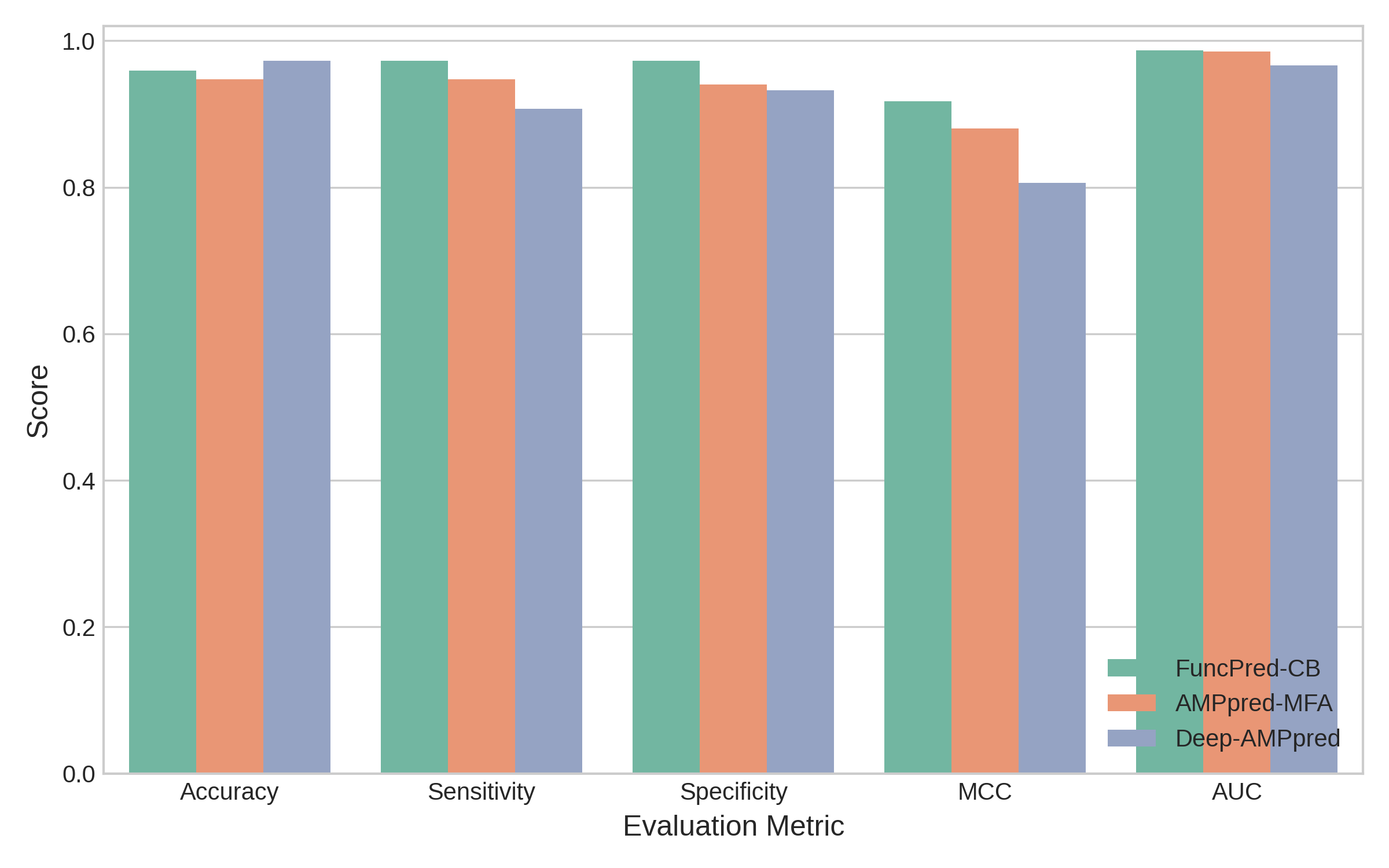


Fig. S3. Performance comparison between FuncPred-CB, AMPpred-MFA and deep-AMPpred on the Dataset_3.

**S3.4 The Impact of Basic Nucleotides on MHA-preconv**

Since the base frequency differences between coding and non-coding regions can reveal structural and functional features of the genome, the data distribution of coding and non-coding regions is plotted as shown in Fig. S4. It can be observed that the frequency of A bases in coding regions is significantly higher than in non-coding regions, while the frequencies of G, C, and T bases are lower in coding regions than in non-coding regions. Therefore, comparing the base frequencies between coding and non-coding regions can serve as an important tool for identifying gene-coding regions, predicting gene functions, and analyzing genomic data. Dataset 2 was used to study the impact of nucleotide composition as a feature on the model. Each complete genome contains hundreds of thousands or millions of bases, and 700 bp fragments were randomly selected from each test genome for measurement, with 5× coverage, repeated 10 times. In Table S1, the model was tested with and without the extraction of nucleotide composition features. It can be observed that including nucleotide composition as a feature improves the model's accuracy, sensitivity, specificity, and other metrics in predictions across different genomes.


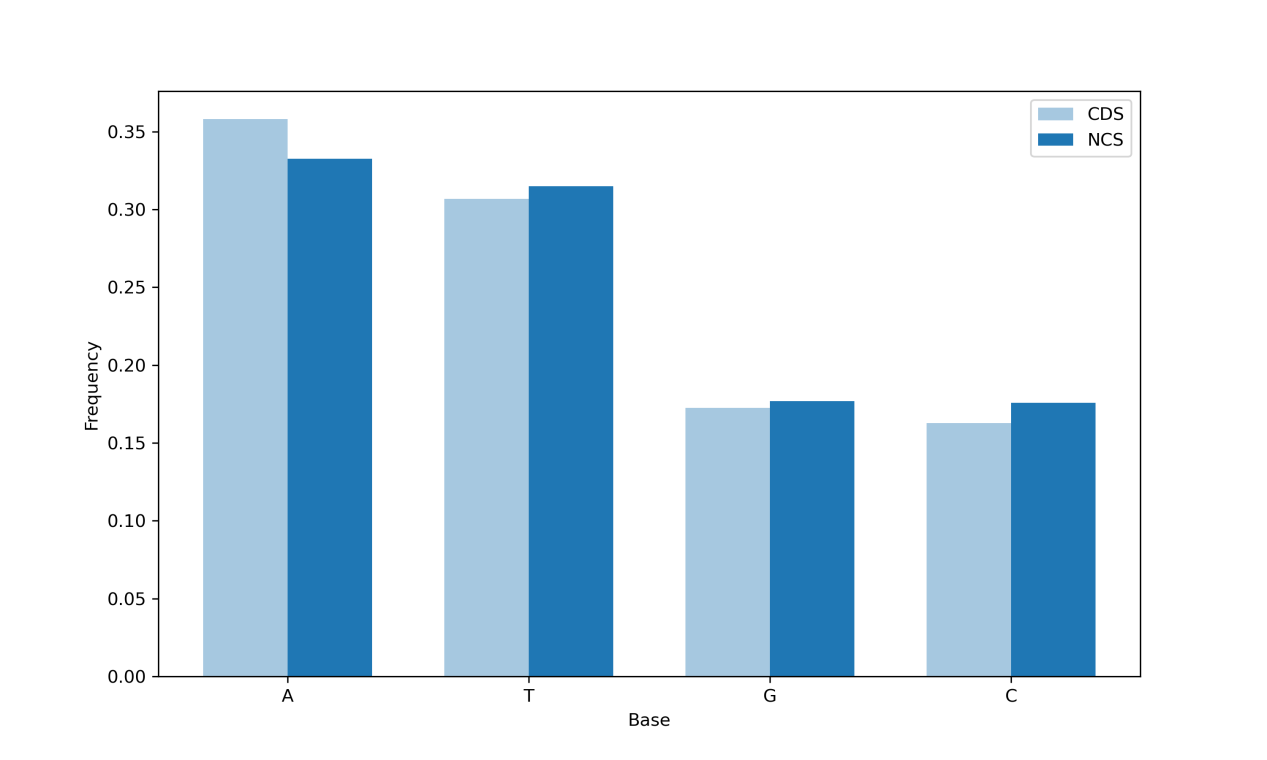


Fig. S4. Nucleotide Distribution in Coding and Non-Coding Regions.
Table. S1. Comparison of MHA-preconv Model Performance Based on Basic Nucleotide Composition Features.

|  |  | base_composition |  |  | nobase_composition |  |
| --- | --- | --- | --- | --- | --- | --- |
| Genome ID | Acc | Sn | Sp | Acc | Sn | Sp |
| NC_002516.1 | 97.33 | 95.33 | 98.04 | 94.26 | 92.40 | 94.92 |
| NC_000909.1 | 97.24 | 95.16 | 97.97 | 95.70 | 95.50 | 97.35 |
| NC_007426.1 | 97.18 | 94.98 | 98.04 | 93.32 | 92.83 | 93.49 |
| NC_002528.1 | 97.30 | 97.23 | 95.43 | 85.79 | 76.33 | 85.07 |
| NC_007164.1 | 97.17 | 98.02 | 97.04 | 96.70 | 94.56 | 97.71 |
| NC_002932.1 | 97.56 | 96.83 | 97.24 | 93.63 | 95.42 | 93.00 |
| NC_000921.1 | 97.30 | 98.22 | 96.84 | 95.32 | 94.21 | 97.38 |
| NC_007577.1 | 97.43 | 94.63 | 97.33 | 88.37 | 91.96 | 78.99 |
| NC_006833.1 | 97.04 | 95.83 | 97.24 | 95.68 | 95.68 | 94.67 |
| NC_006350.1 | 97.29 | 95.07 | 98.07 | 96.35 | 95.94 | 96.50 |

**S3.5 Comparison of CNN, Transformer, and MHA-preconv Models**

Specifically, the MHA-preconv model consists of two distinct sub-architectures: a CNN submodule and a Transformer encoder layer submodule. To evaluate the contribution of each component, we compared the full MHA-preconv model with standalone CNN and Transformer models. The detailed performance metrics are presented in Table. S2. As shown, the integrated CNN-Transformer architecture achieves a 2.56% higher accuracy compared to the standalone CNN model and a 2.1% improvement over the standalone Transformer model. Furthermore, the integrated model consistently outperforms both individual models across other metrics such as sensitivity and overall predictive performance.

Table. S2. Performance Comparison of Different Modules within the MHA-preconv Model.

|  | CNN | Transformer | CNN-Transformer |
| --- | --- | --- | --- |
| Accuracy(%) | 94.83 | 95.29 | **97.39** |
| Sn(%) | 89.57 | 95.08 | **98.17** |
| Sp(%) | **97.45** | 95.40 | 96.47 |
| HM(%) | 93.34 | 95.24 | **95.83** |
| Precision(%) | 94.59 | 91.13 | **94.96** |
| F1 score(%) | 92.01 | 93.06 | **93.82** |
| MCC(%) | 88.27 | 89.55 | **90.82** |

**S3.6 Impact of Different CNN Layers on the Performance of MHA-preconv**

To further investigate the impact of CNN depth on model performance, we conducted a series of comparative experiments using architectures with 1 to 5 convolutional layers. The results are summarized in Table. S3. The findings indicate that the model achieved optimal performance with two convolutional layers, reaching an accuracy of 96.78%. Increasing the number of CNN layers beyond this point did not lead to further performance improvements; in fact, it caused a decline in accuracy in some configurations. This degradation may be attributed to the introduction of redundancy or overfitting due to the excessive depth of the convolutional structure, which can interfere with the effective learning of local features. Therefore, in this task, a moderate CNN depth is more conducive to robust feature extraction and improved model generalization.

Table. S3. Performance Comparison of Different Convolutional Layer Configurations in the CNN Model.

|  | 1conv | 2conv | 3conv | 4conv | 5conv |
| --- | --- | --- | --- | --- | --- |
| Accuracy(%) | 93.79 | **96.78** | 89.93 | 76.46 | 73.05 |
| Sn(%) | 81.89 | **92.13** | 89.37 | 74.21 | 35.43 |
| Sp(%) | **99.71** | 98.43 | 90.21 | 77.57 | 91.77 |
| HM(%) | 89.92 | **94.76** | 89.79 | 75.85 | 51.13 |
| Precision(%) | **99.28** | 96.70 | 81.95 | 62.21 | 68.18 |
| F1 score(%) | 89.75 | **94.35** | 85.50 | 67.68 | 46.63 |
| MCC(%) | 86.17 | **91.71** | 77.98 | 49.87 | 33.90 |

**S3.7 Impact of Different Encoder Layers on the Performance of MHA-preconv**

In neural network models, the number of encoder layers can significantly impact performance. A greater number of encoder layers increases model complexity, leading to longer training times and higher computational demands. In some cases, adding more encoder layers can improve performance by enabling the model to capture more complex features. However, this improvement is not always linear and may plateau or even decline beyond a certain point. To investigate this, we conducted experiments with 0 to 5 encoder layers in the model. As shown in Table.S4, using a single encoder layer achieved the highest accuracy. Considering all performance metrics comprehensively, the model with one encoder layer yielded the best overall performance while also requiring the shortest training and testing time.

Table. S4. Performance Comparison with Different Numbers of Encoder Layers.

|  | 1encoder | 2encoder | 3encoder | 4encoder | 5encoder |
| --- | --- | --- | --- | --- | --- |
| Accuracy(%) | **96.98** | 96.40 | 86.98 | 96.34 | 96.14 |
| Sn(%) | **93.90** | 93.31 | 61.02 | 92.52 | 90.35 |
| Sp(%) | 97.26 | 97.94 | **99.90** | 98.24 | 99.02 |
| HM(%) | **95.60** | 95.57 | 75.77 | 95.29 | 94.49 |
| Precision(%) | 99.58 | 95.76 | **99.68** | 96.31 | 97.87 |
| F1 score(%) | 94.17 | **94.52** | 75.70 | 94.38 | 93.96 |
| MCC(%) | 91.29 | **91.86** | 71.29 | 91.70 | 91.29 |

**S3.8 Impact of the Bi-LSTM Module on FuncPred-CB**

To further validate the role of the Bi-LSTM module in functional peptide recognition, we compared token-level feature heatmaps generated by two architectures: CNN-only (Fig. S5) and CNN-BiLSTM (Fig. S6). As illustrated in the figures, the CNN model tends to focus on a limited number of amino acid positions, with a relatively localized distribution of feature activation. In contrast, the inclusion of the Bi-LSTM module enables the model to assign higher response weights across a broader range of sequence positions—particularly in the middle and downstream regions where several key residues are located. This shift indicates that the Bi-LSTM module effectively enhances the model’s ability to capture contextual dependencies, enabling it to learn long-range sequence relationships and generate more comprehensive feature representations. Experimental results demonstrate that the dual-channel structure significantly improves overall performance in both ACP and AMP prediction tasks, confirming the effectiveness of Bi-LSTM in modeling the biological semantics of peptide sequences.


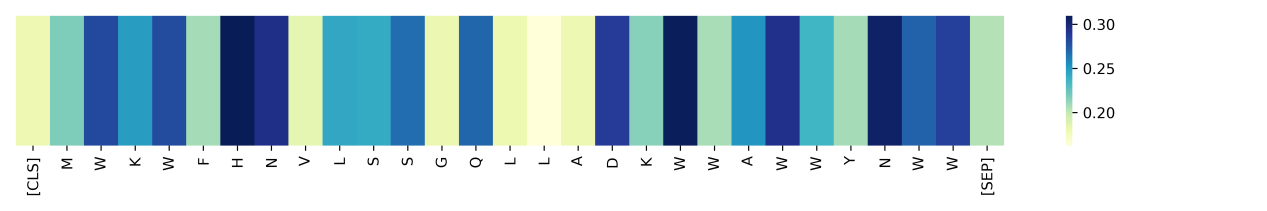


Fig. S5. Amino Acid Attention Distribution for Coding and Non-Coding Regions under the CNN Architecture.


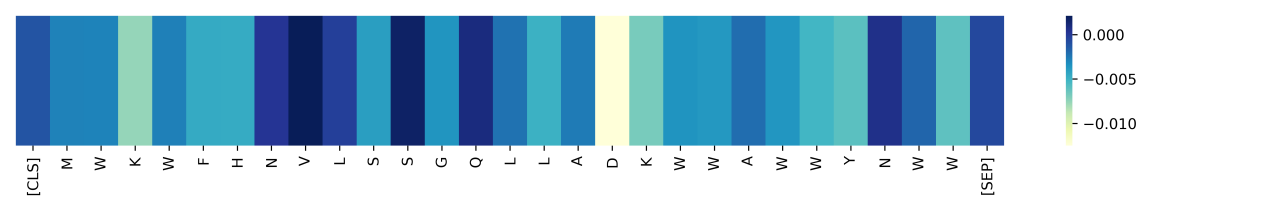


Fig. S6. Amino Acid Attention Distribution for Coding and Non-Coding Regions under the CNN-BiLSTM Architecture.

**S3.9 Comparative performance metrics across different modules**

We conducted comparative experiments on top of the GCN baseline, and the results are summarized in Table S5. Although the GCN module achieves competitive accuracy, it is consistently outperformed by the CNN-BiLSTM module across all evaluation metrics. Further head-to-head comparison between Bert-GCN and Bert-CNN-BiLSTM reveals that our model surpasses Bert-GCN on every metric, indicating a fundamental semantic mismatch between the “graph structure” imposed by GCN and the intrinsic nature of peptide sequences. The biological activity of peptides primarily arises from contiguous or short-range conserved motifs; yet, to apply graph convolution, GCN is forced to convert the entire sequence into a fully connected or k-NN graph, rendering ~90 % of the edges “noise edges.” These spurious connections are repeatedly amplified during message passing, diluting the true signal of critical residues. In contrast, BiLSTM explicitly models sequential order through forward and backward hidden states, exhibiting linear sensitivity to positional changes, whereas GCN must rely on positional encodings that are far less effective than the explicit state transitions of LSTM. Collectively, BERT’s global contextualization, CNN’s local motif detection, and BiLSTM’s mid-range sequential modeling provide complementary inductive biases that are essential for accurate functional-peptide prediction.

|  | Accuracy(%) | Sn(%) | Sp(%) | MCC(%) | AUC(%) |
| --- | --- | --- | --- | --- | --- |
| Bert-CNN-Bi LSTM | 92.49 | 91.19 | 93.78 | 86.72 | 94.58 |
| Bert | 82.56 | 83.42 | 89.71 | 75.39 | 85.90 |
| CNN | 76.95 | 77.32 | 72.45 | 54.33 | 81.24 |
| Bi LSTM | 77.57 | 74.26 | 79.11 | 55.76 | 78.29 |
| GCN | 77.94 | 78.87 | 77.36 | 56.90 | 78.68 |
| CNN-Bi LSTM | 88.34 | 83.42 | 93.26 | 80.34 | 85.72 |
| Bert-GCN | 89.63 | 85.11 | 90.74 | 75.41 | 85.25 |

**S3.10 Summary of Predicted ACP and AMP Sequences with Putative Activity**

| **Sequence** | **Name** | **Length** | **Nature** | **Origin** | **Cancer Type** |
| --- | --- | --- | --- | --- | --- |
| MWKWFHNVLSSGQLLADKWWAWWYNWW | Pep27anal5 | 27 | Anticancer | S. pneumoniae | Leukemia Cancer、Blood Cancer、Gastric Cancer、Breast Cancer |
| FALAKLAKKAKAKLKKALKAL | FLAK05 AM | 21 | Anticancer | FLAK peptides | Breast Cancer、Colon Cancer、Skin Cancer、Lung Cancer、Prostate Cancer、Cervical Cancer |
| FAKKLLAKALKL | FLAG26-D3 | 12 | Anticancer | FLAK peptides | Breast Cancer、Colon Cancer、Skin Cancer、Lung Cancer、Prostate Cancer、Cervical Cancer |
| VAKKLAKLAKKLAKLALAL | FLAK 25 AM V | 19 | Anticancer | FLAK peptides | Breast Cancer、Skin Cancer |
| FAKKLAKLAKKLLAL | FLAK43 AM | 15 | Anticancer | FLAK peptides | Breast Cancer、Colon Cancer、Skin Cancer、Lung Cancer、Prostate Cancer、Cervical Cancer |
| FAKKLKKLAKLAKKL | FLAK71 | 15 | Anticancer | FLAK peptides | Breast Cancer、Colon Cancer、Skin Cancer、Lung Cancer、Prostate Cancer、Cervical Cancer |
| LLRHVVKILEKYL | Temporin-La | 13 | Anticancer | Temporins family | Colorectal Cancer、Liver Cancer、Cervical Cancer 、Gastric Cancer、Lung Cancer、Renal Cancer、Breast Cancer |
| GLFDVIKKVASVIKKL | 13 | 16 | Antimicrobial and Anticancer | Amphibian skin secretions | Leukemia Cancer、Lung Cancer、Colon Cancer、Brain Tumor、Skin Cancer、Ovarian Cancer、Liver Cancer、Prostate Cancer、Breast Cancer |
| FKCRRWQWRMKK | LfcinB | 12 | Anticancer | Bovine lactoferrin (Lf-B) | Skin Cancer、Colon Cancer、Lymphoma Cancer |
| FKCRRWQWRMKK | LfcinB | 12 | Antimicrobial and Anticancer | Bovine lactoferrin (Lf-B) | Colon Cancer、Breast Cancer、Brain Tumor |
| FAKLLKLAAKKLL | FLAK 50E | 13 | Anticancer | FLAK peptides | Breast Cancer、Colon Cancer、Skin Cancer、Lung Cancer、Prostate Cancer、Cervical Cancer |
| VALALKALKKALKKLKKALKKAL | KAL V、Hecate AM V | 23 | Anticancer | FLAK peptides | Breast Cancer、Colon Cancer、Skin Cancer、Lung Cancer、Prostate Cancer、Cervical Cancer |
| GLLSVLGSVAKHVLPHVVPVIAEKL | Caerin 1.10 | 25 | Anticancer | Magnificent treefrog Litoria splendida, Australia | Lung Cancer |
| FALAAKALKKLAKKLKKLAKKAL | FLAK01 AM | 23 | Anticancer | FLAK peptides | Breast Cancer、Colon Cancer、Skin Cancer、Lung Cancer、Prostate Cancer、Cervical Cancer |
| LPKWKVFKKIEKVGRNIRNGIVKAGPAIAVLGEAKALG | LSB-37 | 38 | Anticancer | FLAK peptides | Breast Cancer、Colon Cancer、Skin Cancer、Lung Cancer、Prostate Cancer、Cervical Cancer |
| KAAKKAWKAAKKAWKAAKKAA | Z7 | 21 | Anticancer | Synthetic | Skin Cancer、Breast Cancer、Colon Cancer |
| GLFKVIKKVASVIGGL | 22 | 16 | Antimicrobial and Anticancer | Amphibian skin secretions | Leukemia Cancer、Lung Cancer、Colon Cancer、Brain Tumor、Skin Cancer、Ovarian Cancer、Liver Cancer、Prostate Cancer、Breast Cancer |
| KWWKKAAKAAKKAAKAAKKWA | Z6 | 21 | Anticancer | Synthetic | Skin Cancer、Breast Cancer、Colon Cancer |
| FAKKLAKLALKLAKL | FLAK51 | 15 | Anticancer | FLAK peptides | Breast Cancer、Colon Cancer、Skin Cancer、Lung Cancer、Prostate Cancer、Cervical Cancer |
| FAKKLAKKLKKLAKKLAKLALAL | SHIVA 10 AC | 23 | Anticancer | FLAK peptides | Breast Cancer、Colon Cancer、Skin Cancer、Lung Cancer、Prostate Cancer、Cervical Cancer |
| KWKSFAKTFKSAKKTVAHTALKAISS | L6A/L17A | 26 | Anticancer | PeptideV13K | Lung Cancer、Skin Cancer、Breast Cancer、Colorectal Cancer、Rhabdomyosarcoma Cancer、Cervical Cancer |
| FAKLLAKLAKKIL | FLAK50 T3 | 13 | Anticancer | FLAK peptides | Breast Cancer、Colon Cancer、Skin Cancer、Lung Cancer、Prostate Cancer、Cervical Cancer |
| FAKLLAKFLKKAL | FLAK50 Q3 | 13 | Anticancer | FLAK peptides | Breast Cancer、Colon Cancer、Skin Cancer、Lung Cancer、Prostate Cancer、Cervical Cancer |
| GLPVCGETCAGGTCNTPGCSCSWPICTRN | Vaby A | 29 | Anticancer | African, the Ethiopian highlands, Viola abyssinica | Fibrosarcoma |
| KILRGVAKKILRTFLRRISKDILTGKK | C7A | 27 | Anticancer | NK-2 variants | Skin Cancer |
| VAKKFAKKFKKFAKKFAKFAFAF | D2A21V | 23 | Anticancer | FLAK peptides | Breast Cancer、Colon Cancer、Skin Cancer |
| LLGMIPLAISAISALSKL | Phylloseptin-L1 | 32 | Anticancer | Hylomantis lemur (Hylidae: Phyllomedusinae | Colorectal Cancer |
| GLFAVIKKVAAVIRRL | 18 | 16 | Antimicrobial and Anticancer | Amphibian skin secretions | Leukemia Cancer、Lung Cancer、Colon Cancer、Brain Tumor、Skin Cancer、Ovarian Cancer、Liver Cancer、Prostate Cancer、Breast Cancer |
| FLGALFHALSKLL | PTP-7a | 13 | Anticancer | Synthetic peptide | Lung Cancer |
| FAKLLAKLAKKAL | FLAK50C | 13 | Anticancer | FLAK peptides | Breast Cancer、Colon Cancer、Skin Cancer、Lung Cancer、Prostate Cancer、Cervical Cancer |
| ILPWKWPWWPWRR | Indolicidin、Indolocidin-ac | 13 | Anticancer | FLAK peptides | Breast Cancer、Colon Cancer、Skin Cancer、Lung Cancer、Prostate Cancer、Cervical Cancer |
| FKVKFKVKVK | KSL-7 | 10 | Anticancer | FLAK peptides | Breast Cancer、Colon Cancer、Skin Cancer、Lung Cancer、Prostate Cancer、Cervical Cancer |
| FLGALFKALSHLL | PTP-7b | 13 | Anticancer | Synthetic peptide | Lung Cancer |
| FAKLLAKLAK | FLAK50 T1 | 10 | Anticancer | FLAK peptides | Breast Cancer |
| LGGIVSAVKKIVDFLG | retro | 16 | Antimicrobial and Anticancer | Amphibian skin secretions | Leukemia Cancer、Lung Cancer、Colon Cancer、Brain Tumor、Skin Cancer、Ovarian Cancer、Liver Cancer、Prostate Cancer、Breast Cancer |
| AAKKWAKAKWAKAKKWAKAA | Z10 | 21 | Anticancer | Synthetic | Skin Cancer、Breast Cancer、Colon Cancer |
| KWKSFLKTFKSLKKTVLHTLLKLISS | A12L/A20L/A23L | 26 | Anticancer | PeptideV13K | Lung Cancer、Skin Cancer、Breast Cancer、Colorectal Cancer、Rhabdomyosarcoma Cancer、Cervical Cancer |
| FAKKLAKKLAKLL | FLAK 56 | 13 | Anticancer | FLAK peptides | Breast Cancer 、Colon Cancer、Skin Cancer、Lung Cancer、Prostate Cancer、Cervical Cancer |
| GLFAVIKKVASVIKGL | 15 | 16 | Antimicrobial and Anticancer | Amphibian skin secretions | Leukemia Cancer、Lung Cancer、Colon Cancer、Brain Tumor、Skin Cancer、Ovarian Cancer、Liver Cancer、Prostate Cancer、Breast Cancer |

| **Sequence：** | **Name** | **Length** | **Nature** | **Origin** | **Cancer Type** | **Assay** |
| --- | --- | --- | --- | --- | --- | --- |
| GLWSKIKEVGKEAAKAAAKAAGKAALGAVSEAV | Adenoregulin | 33 | Antimicrobial | South American frog Phyllomedusa bicolor | Prostate Cancer、Liver Cancer、Breast Cancer、Lymphoma Cancer | LDH leakage assay |
| KWKFKKIPKFLHLAKKF | P18 | 17 | Antimicrobial | Ceropin A | Breast Cancer、Blood Cancer、Leukemia Cancer | MTT/MTS |
| GIIKKIIKKIIKKIIKKI | Short α-helical peptides | 18 | Antimicrobial | Alpha-helical proteins | Cervical Cancer、Leukemia Cancer | MTT/MTS |
| GLFDIAKKVIGVIGSL | Aurein2.6 | 16 | Antimicrobial | Southern bell frog | Breast Cancer、Leukemia Cancer、Lung Cancer、Colon Cancer、Brain Tumor、Skin Cancer、Ovarian Cancer、Renal Cancer、Prostate Cancer |  |
| KILRGVAKKIMRTFLRRILTGKK | C7A-Δ | 24 | Antimicrobial | Synthetic peptide | Skin Cancer | MTT/MTS |
| GLFDVIKKVASVIKKL | 13 | 16 | Antimicrobial and Anticancer | Amphibian skin secretions | Leukemia Cancer、Lung Cancer、Colon Cancer、Brain Tumor、Skin Cancer、Ovarian Cancer、Liver Cancer、Prostate Cancer、Breast Cancer | Sulforhodamine B assay |
| FKCRRWQWRMKK | LfcinB | 12 | Antimicrobial and Anticancer | Bovine lactoferrin (Lf-B) | Colon Cancer、Breast Cancer、Brain Tumor | MTT/MTS |
| KWKLFKKIPKFLH | C-10 | 13 | Antimicrobial | Ceropin A | Breast Cancer、Blood Cancer、Leukemia Cancer | MTT/MTS |
| VNWKKILGKIIKVVK | LL-III、LL-III/8、LL-III/9、LL-III/12、LL-III/37 | 15 | Antimicrobial | Lasioglossin III and its analogs | Cervical Cancer、Colon Cancer、Leukemia Cancer | MTT/MTS |
| KWKLFKKIPFLHLAKKF | C-1 | 17 | Antimicrobial | Ceropin A | Breast Cancer、Blood Cancer、Leukemia Cancer | MTT/MTS |
| GKFMSLLKHILK | HAL-2/24 | 12 | Antimicrobial | Halictines and their analogs | Cervical Cancer 、Colon Cancer、Leukemia Cancer | MTT/MTS |
| GMWSKILGHLIR | HAL-1、HAL-1/22、HAL-1/29 | 12 | Antimicrobial | Halictines and their analogs | Cervical Cancer、Colon Cancer、Leukemia Cancer | MTT/MTS |
| KWKLFKKIPKFLHLAK | C-4 | 16 | Antimicrobial | Ceropin A | Blood Cancer、Leukemia Cancer | MTT/MTS |
| FFHHIFRGIVHVGKTIHRLVTG | Piscidin 1 | 22 | Antimicrobial | Hybrid striped bass (Morone saxatilis x M. chrysops) | Breast Cancer |  |
| PAWRKAFRWAKRMLKKAA | L12 | 18 | Antimicrobial | Lactoferrin | Skin Cancer、Breast Cancer、Colon Cancer | MTT/MTS |
| WKKIPKFLHLAKKF | N-5 | 14 | Antimicrobial | Ceropin A | Breast Cancer、Blood Cancer、Leukemia Cancer | MTT/MTS |
| GLFKVIKKVASVIGGL | 22 | 16 | Antimicrobial and Anticancer | Amphibian skin secretions | Leukemia Cancer、Lung Cancer、Colon Cancer、Brain Tumor、Skin Cancer、Ovarian Cancer、Liver Cancer、Prostate Cancer、Breast Cancer | Sulforhodamine B |
| GLFDIIKKIAESF | Aurein1.2 | 13 | Antimicrobial | Southern bell frog | Breast Cancer、Leukemia Cancer、Lung Cancer、Colon Cancer、Brain Tumor、Skin Cancer、Ovarian Cancer、Renal Cancer、Prostate Cancer |  |
| GFFALIPKIISSPLFKTLLSAVGSALS | Pardaxin-6 | 27 | Antimicrobial | Epinephelus nebulosus | Fibrosarcoma、Cervical Cancer | MTT/MTS |
| SIGAKILGGVKTFFKGALKELASTYLQ | maximin5 | 27 | Antimicrobial | Bombina maxima | Leukemia Cancer、Bladder Cancer | MTT/MTS |
| GKWMSLLKHWLK | HAL-2/19 | 12 | Antimicrobial | Halictines and their analogs | Cervical Cancer、Colon Cancer、Leukemia Cancer | MTT/MTS |
| KTCENLADTY | Sesquin | 10 | Antimicrobial | Seeds, Vigna sesquipedalis, ground bean | Colorectal Cancer |  |
| FLKWLFKWAKK | Gaegurin peptides SEQID NO: 2 | 11 | Antimicrobial | Korean frog Rana rugosa | Skin Cancer、Lung Cancer、Ovarian Cancer、Gastric Cancer、Prostate Cancer、Renal Cancer、Colon Cancer | MTT/MTS |
| KWKLFKKIPLAKKF | C-7 | 14 | Antibacterial | Ceropin A | Breast Cancer、Blood Cancer、Leukemia Cancer | MTT/MTS |
| GLFDIVKKIAGHIAGSI | Aurein 3.1 | 17 | Antibacterial | Southern bell frog | Breast Cancer、Leukemia Cancer、Lung Cancer、Colon Cancer、Brain Tumor、Skin Cancer、Ovarian Cancer、Renal Cancer、Prostate Cancer |  |
| GIIKKIIKKI | Short α-helical peptides | 10 | Antibacterial | Alpha-helical proteins | Cervical Cancer、Leukemia Cancer | MTT/MTS |
| KILRGVAKKIMRTFLRRISKDILTGKK | C7A | 27 | Antibacterial | Synthetic peptide | Skin Cancer | MTT/MTS |
| GMWKKILGKLIR | HAL-1/10 | 12 | Antibacterial | Halictines and their analogs | Cervical Cancer、Colon Cancer、Leukemia Cancer | MTT/MTS |
| FVDLKKIANIINSIF | Tempoprin-1CEa | 15 | Antibacterial | Chinese brown frog Rana chensinensis. | Breast Cancer |  |
| GLFAVIKKVAAVIRRL | 18 | 16 | Antimicrobial and Anticancer | Amphibian skin secretions | Leukemia Cancer、Lung Cancer、Colon Cancer、Brain Tumor、Skin Cancer、Ovarian Cancer、Liver Cancer、Prostate Cancer、Breast Cancer | Sulforhodamine B |
| GFKMALKLLKKVL | MAC1/9、MAC1/10 | 13 | Antimicrobial | Macropins and their analogs | Cervical Cancer、Colon Cancer、Leukemia Cancer | MTT/MTS |
| FKCRRWQWRMKKLGA | Lactoferricin B | 15 | Antimicrobial | Temporins family | Brain Tumor | MTT/MTS |
| KWKLFKKIGIGAFLHSAKKF | P3 | 20 | Antimicrobial | Ceropin A-Magainin 2 | Lung Cancer | MTT/MTS |
| LGGIVSAVKKIVDFLG | retro | 16 | Antimicrobial and Anticancer | Amphibian skin secretions | Leukemia Cancer、Lung Cancer、Colon Cancer、Brain Tumor、Skin Cancer、Ovarian Cancer、Liver Cancer、Prostate Cancer、Breast Cancer | Sulforhodamine B |
| WFKKIPKFLHLAKKF | N-4 | 15 | Antimicrobial | Ceropin A | Breast Cancer、Blood Cancer、Leukemia Cancer | MTT/MTS |
| KWKLFKKIPKFLHSAKKF | CA-MA-P、CA-MA2 | 18 | Antimicrobial | Ceropin A、Ceropin-Magainin hybrid peptides | Breast Cancer、Blood Cancer、Leukemia Cancer | MTT/MTS |
| FLPLIGRVLSGIL | Temporin A | 13 | Antimicrobial | Frog Rana temporaria | Lymphoma Cancer | MTT/MTS |
| PAWFKARRWAWRMLKKAA | L4 | 15 | Antimicrobial | Lactoferrin | Skin Cancer、Breast Cancer、Colon Cancer | MTT/MTS |
| GLFAVIKKVASVIKGL | 15 | 16 | Antimicrobial and Anticancer | Amphibian skin secretions | Leukemia Cancer、Lung Cancer、Colon Cancer、Brain Tumor、Skin Cancer、Ovarian Cancer、Liver Cancer、Prostate Cancer、Breast Cancer | Sulforhodamine B |

**References**

[1]Koonin E V. Orthologs, paralogs, and evolutionary genomics[J]. Annu. Rev. Genet., 2005, 39(1): 309-338.

[2]Fickett JW, Tung CS. Assessment of protein coding measures. Nucleic Acids Res. 1992 Dec 25;20(24):6441-50.

[3]Teng W, Liao B, Chen M, Shu W. Genomic Legacies of Ancient Adaptation Illuminate GC-Content Evolution in Bacteria. Microbiol Spectr. 2023 Feb 14;11(1):e0214522.

[4]Warren AS, Setubal JC. The Genome Reverse Compiler: an explorative annotation tool. BMC Bioinformatics. 2009 Jan 27;10:35.

[5]Hoff KJ, Lingner T, Meinicke P, Tech M. Orphelia: predicting genes in metagenomic sequencing reads. Nucleic Acids Res. 2009 Jul;37(Web Server issue):W101-5.

[6]El Allali A, Rose JR. MGC: a metagenomic gene caller. BMC Bioinformatics. 2013;14 Suppl 9(Suppl 9):S6.

[7]O'Leary N A, Wright M W, Brister J R, et al. Reference sequence (RefSeq) database at NCBI: current status, taxonomic expansion, and functional annotation[J]. Nucleic acids research, 2016, 44(D1): D733-D745.

[8]Meyer F, Lesker TR, Koslicki D, Fritz A, Gurevich A, Darling AE, Sczyrba A, Bremges A, McHardy AC. Tutorial: assessing metagenomics software with the CAMI benchmarking toolkit. Nat Protoc. 2021 Apr;16(4):1785-1801.

[9]Sharon I, Morowitz M J, Thomas B C, et al. Time series community genomics analysis reveals rapid shifts in bacterial species, strains, and phage during infant gut colonization[J]. Genome research, 2013, 23(1): 111-120.

[10]Jian Ye, Scott McGinnis, Thomas L. Madden, BLAST: improvements for better sequence analysis, Nucleic Acids Research, Volume 34, Issue suppl_2, 2006(7): W6–W9.

[11]Benson D A, Karsch‐Mizrachi I, Lipman D J, et al. GenBank: update[J]. Nucleic acids research, 2004, 32(suppl_1): D23-D26.

[12]Agrawal P, Bhagat D, Mahalwal M, et al. AntiCP 2.0: an updated model for predicting anticancer peptides[J]. Briefings in bioinformatics, 2021, 22(3): bbaa153.

[13]Novković M, Simunić J, Bojović V, et al. DADP: the database of anuran defense peptides[J]. Bioinformatics, 2012, 28(10): 1406-1407.

[14]Waghu F H, Barai R S, Gurung P, et al. CAMPR3: a database on sequences, structures and signatures of antimicrobial peptides[J]. Nucleic acids research, 2016, 44(D1): D1094-D1097.

[15]Wang Z, Wang G. APD: the antimicrobial peptide database[J]. Nucleic acids research, 2004, 32(suppl_1): D590-D592.

[16]Wang G, Li X, Wang Z. APD2: the updated antimicrobial peptide database and its application in peptide design[J]. Nucleic acids research, 2009, 37(suppl_1): D933-D937.

[17]Tyagi A, Tuknait A, Anand P, et al. CancerPPD: a database of anticancer peptides and proteins[J]. Nucleic acids research, 2015, 43(D1): D837-D843.

[18]UniProt Consortium. UniProt: a hub for protein information[J]. Nucleic acids research, 2015, 43(D1): D204-D212.

[19]Gasteiger E, Jung E, Bairoch A. SWISS-PROT: connecting biomolecular knowledge via a protein database[J]. Current issues in molecular biology, 2001, 3(3): 47-55.

[20]Ma, Yue, et al. "Identification of antimicrobial peptides from the human gut microbiome using deep learning." Nature Biotechnology 40.6 (2022): 921-931.

[21]Wang G, Li X, Wang Z. APD3: the antimicrobial peptide database as a tool for research and education[J]. Nucleic acids research, 2016, 44(D1): D1087-D1093.

[22]Zhao X, Wu H, Lu H, et al. LAMP: a database linking antimicrobial peptides[J]. PloS one, 2013, 8(6): e66557.
